# Evaluation of the chemopreventive potentials of ezetimibe and aspirin in a novel mouse model of gallbladder preneoplasia

**DOI:** 10.1002/1878-0261.12766

**Published:** 2020-09-17

**Authors:** Lorena Rosa, Lorena Lobos‐González, Natalia Muñoz‐Durango, Patricia García, Carolina Bizama, Natalia Gómez, Ximena González, Ignacio A. Wichmann, Nicolás Saavedra, Francisca Guevara, Jaime Villegas, Marco Arrese, Catterina Ferreccio, Alexis M. Kalergis, Juan Francisco Miquel, Jaime A. Espinoza, Juan C. Roa

**Affiliations:** ^1^ Doctorado en Ciencias mención Biología Celular y Molecular Aplicada Universidad de La Frontera Temuco Chile; ^2^ Departamento de Patología Facultad de Medicina Pontificia Universidad Católica de Chile Santiago Chile; ^3^ Centro de Medicina Regenerativa Facultad de Medicina‐Clínica Alemana Universidad del Desarrollo Santiago Chile; ^4^ Advanced Center for Chronic Diseases (ACCDiS) Santiago Chile; ^5^ Millennium Institute of Immunology and Immunotherapy (IMII) Santiago Chile; ^6^ Departmento de Genética Molecular y Microbiología Facultad de Ciencias Biológicas Pontificia Universidad Católica de Chile Santiago Chile; ^7^ Departamento de Hematología‐Oncología Facultad de Medicina Pontificia Universidad Católica de Chile Santiago Chile; ^8^ Departamento de Ciencias Básicas Centro de Biología Molecular y Farmacogenética BIOREN Universidad de La Frontera Temuco Chile; ^9^ Fundación Ciencia & Vida Santiago Chile; ^10^ Centro de Medicina Veterinaria Facultad de Ciencias de la Vida Universidad Andrés Bello Santiago Chile; ^11^ Departamento de Gastroenterología Facultad de Medicina Pontificia Universidad Católica de Chile Santiago Chile; ^12^ Departamento de Salud Publica Facultad de Medicina Pontificia Universidad Católica de Chile Santiago Chile; ^13^ Departmento de Endocrinología Facultad de Medicina Pontificia Universidad Católica de Chile Santiago Chile; ^14^ SciLifeLab Division of Genome Biology Department of Medical Biochemistry and Biophysics Karolinska Institutet Stockholm Sweden

**Keywords:** chronic inflammation, dysplasia, gallbladder cancer, gallstones, lithogenic diet, metaplasia

## Abstract

Gallbladder stones (cholecystolithiasis) are the main risk factor for gallbladder cancer (GBC), a lethal biliary malignancy with poor survival rates worldwide. Gallbladder stones are thought to damage the gallbladder epithelium and trigger chronic inflammation. Preneoplastic lesions that arise in such an inflammatory microenvironment can eventually develop into invasive carcinoma, through mechanisms that are not fully understood. Here, we developed a novel gallbladder preneoplasia mouse model through the administration of two lithogenic diets (a low‐ or a high‐cholesterol diet) in wild‐type C57BL/6 mice over a period of 9 months. Additionally, we evaluated the chemopreventive potentials of the anti‐inflammatory drug aspirin and the cholesterol absorption inhibitor ezetimibe. Both lithogenic diets induced early formation of gallbladder stones, together with extensive inflammatory changes and widespread induction of metaplasia, an epithelial adaptation to tissue injury. Dysplastic lesions were presented only in mice fed with high‐cholesterol diet (62.5%) in late stages (9th month), and no invasive carcinoma was observed at any stage. The cholesterol absorption inhibitor ezetimibe inhibited gallbladder stone formation and completely prevented the onset of metaplasia and dysplasia in both lithogenic diets, whereas aspirin partially reduced metaplasia development only in the low‐cholesterol diet setting. This model recapitulates several of the structural and inflammatory findings observed in human cholecystolithiasic gallbladders, making it relevant for the study of gallbladder carcinogenesis. In addition, our results suggest that the use of cholesterol absorption inhibitors and anti‐inflammatory drugs can be evaluated as chemopreventive strategies to reduce the burden of GBC among high‐risk populations.

## Graphical abstract

A novel mouse model of gallbladder preneoplasia secondary to diet‐induced stones progresses in parallel with intense inflammation. Ezetimibe inhibits stone formation, inflammation, and metaplasia–dysplasia development. Aspirin does not reduce stone formation, however, ameliorates inflammation and preneoplasia onset (but only in a low‐cholesterol diet). This model recapitulates the metaplasia–dysplasia sequence observed in humans and is suitable for gallbladder carcinogenesis research.

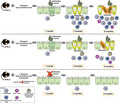

**Abbreviations**
AB/PAS: Alcian blue/periodic acid–Schiff stain
FFPE: formalin‐fixed paraffin‐embedded
GBC: gallbladder cancer
H&E: hematoxylin and eosin stain
IHC: immunohistochemistry
PMN: polymorphonuclear cells
wt/wt: weight/weight

## 1 Introduction

Gallbladder cancer (GBC) is the most common cancer of the biliary tract, with a distinct regional variation worldwide [1]. Highest incidence rates are reported in Bolivia (14.0/100 000), Chile (9.3/100 000), and Thailand (7.4/100 000) [2, 3]. GBC is a highly lethal malignancy, with an overall mean survival of 6 months [1]. Notably, patients diagnosed at T1 stage (carcinoma *in situ*) have a favorable 5‐year survival of 90%, which dramatically drops to 43–65% in pT2 stage (subserosal GBC) and to 5% in advanced GBC, stage at which the tumor has penetrated through the tunica muscularis [4, 5]. Therefore, early detection and new prevention strategies in preneoplastic conditions are important areas for GBC research.

Cholecystolithiasis, or gallbladder stones, is the main risk factor for GBC [1, 6]. Although the association between gallbladder stones and GBC has been known for decades, the precise mechanisms explaining how cholecystolithiasis promotes carcinogenesis remain unknown. From a histopathological point of view, the analysis of gallbladders from cholecystolithiasic patients has shown that the presence of gallbladder stones is often associated with structural changes in the epithelium, thickening of the gallbladder wall, tissue injury, and chronic inflammation [7, 8]. A common feature of cholecystolithiasis is the onset of metaplasia, an epithelial adaptation to tissue injury that is thought to serve as a protective role against chronic damage, either through replacement of lost tissue or by forming barriers better suited to withstand hostile conditions [9]. Although it is not considered an oncogenic stage, metaplasia is generally accepted as a precursor lesion to low‐grade dysplasia, high‐grade dysplasia, and invasive carcinoma [10]. Indeed, metaplasia is found in the majority of gallbladders harboring gallbladder stones [7] and is often associated with the presence of dysplastic foci [11] and invasive carcinomas [12]. In addition, genetic alterations such as TP53 allele loss or mutations can be found in apparently normal epithelia [13], suggesting that genetic changes arise early in the context of cholecystolithiasis. Although the association between metaplasia and cancer predisposition has been observed in various organs [10], including the gallbladder [12], the underlying mechanisms orchestrating the onset of metaplasia and progression to cancer are still not fully understood.

Here, we developed a novel mouse model of gallbladder preneoplasia that recapitulates the metaplasia–dysplasia sequence observed in humans, promoted by cholecystolithiasis and associated with chronic inflammation. Moreover, we tested the effects of two FDA‐approved drugs on the development and progression of preneoplastic lesions: (a) ezetimibe, a cholesterol absorption inhibitor which is known to prevent and dissolve gallbladder stones [14, 15], and (b) aspirin, known for its anti‐inflammatory properties. In our model, ezetimibe prevented the onset of metaplasia and dysplasia by inhibiting gallbladder stone formation. On the other hand, aspirin treatment partially reduced metaplasia development. Our study provides a new animal model of gallbladder preneoplasia and presents preclinical evidence supporting that pharmacological inhibition of cholecystolithiasis and inflammatory processes may serve as chemopreventive strategies to reduce GBC risk in high‐risk populations.

## 2 Materials and methods

### 2.1 Animal experiments

#### 2.1.1 Development of gallbladder preneoplasia mouse model

**Fig. 1:**
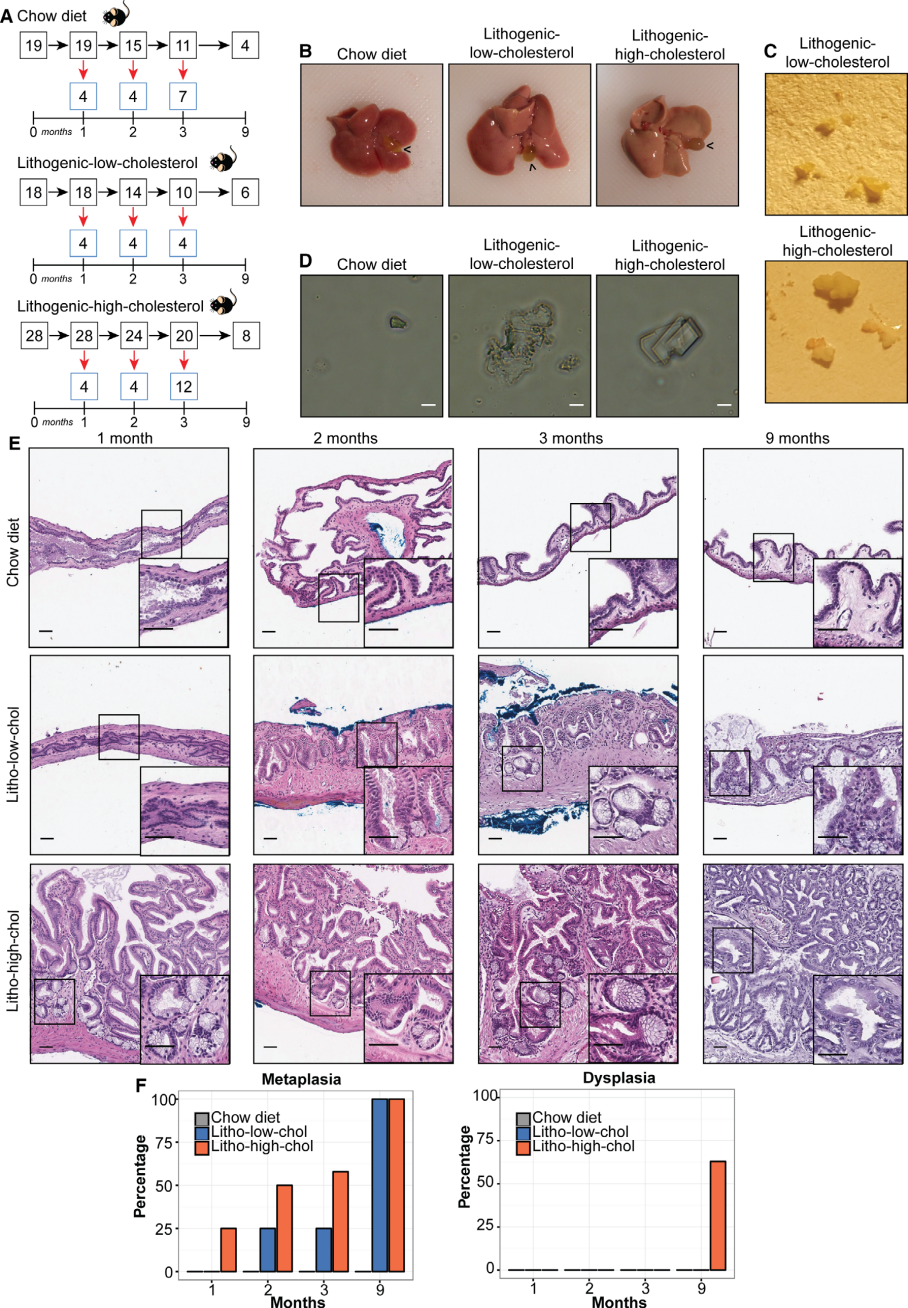
Characterization of a gallbladder preneoplasia mouse model. (A) Experimental design. Three different diet groups (chow, lithogenic low‐cholesterol, and lithogenic high‐cholesterol diets) are shown, indicating the number of mice used at each time point. Red arrows indicate number of mice that were euthanized, and black arrows indicate mice that remained for the following time point. (B) Mice from both lithogenic diets displayed bloated gallbladders (black arrowheads) and steatotic livers. (C) Macroscopic photographs of representative gallbladder stones induced by each lithogenic diet. (D) Light microscopy of bile extracted from mice fed with each diet, showing representative cholesterol crystals present in each group (scale bar, 20 µm). (E) Epithelial alterations of the gallbladder over 9 months of exposure to lithogenic low‐cholesterol (litho‐low‐chol) and lithogenic high‐cholesterol (litho‐high‐chol) diets in H&E‐stained slides. Metaplasia is present in both lithogenic groups at months 2, 3, and 9, and also present at month 1 only in the litho‐high‐chol group (onset magnifications). Scale bar, 200 µm; magnification scale bar, 50 µm. (F) Percentage of metaplasia and dysplasia induced by each diet at each time point. None of the chow animal developed metaplasia neither dysplasia at any time point (*n* = 4 for 1, 2, and 9 months and *n* = 7 for 3 months). Metaplasia was found in 0/4, 1/4, 1/4, and 6/6 for litho‐low‐chol and 1/4, 2/4, 7/12, and 8/8 for litho‐high‐chol at 1, 2, 3, and 9 months; respectively. Dysplasia was only found in 5/8 litho‐high‐chol at 9 months. Sample size for each group at each month was the same as indicated for metaplasia lesion.

Sixty‐five 8‐week‐old male C57BL/6 mice were randomly assigned into three groups and fed one of the following diets: (a) chow diet, *n* = 19 (0.02% weight/weight [wt/wt] cholesterol; Prolab RMH3000; LabDiet, St. Louis, MO, USA); (b) lithogenic high‐cholesterol diet, *n* = 28 (1.25% wt/wt cholesterol, 0.5% wt/wt cholic acid; 57BB mouse diet; Test Diet, St. Louis, MO, USA); and (c) lithogenic low‐cholesterol diet, *n* = 18 (1 : 1, chow diet: lithogenic high‐cholesterol diet mix). We added the lithogenic low‐cholesterol group to evaluate the effects of a moderate increase in cholesterol levels, between those observed in chow and lithogenic high‐cholesterol diets. These mice were maintained with water and their respective diets *ad libitum*in a pathogen‐free environment. Four randomly selected mice from each study group were euthanized at months 1 and 2 to confirm development of gallbladder stones in both lithogenic groups within this timeframe, as reported in previous studies [14, 15]. Next, 4–12 mice from each group were euthanized at months 3 and 9 (Fig. 1A). Gallbladder, liver, spleen, and plasma samples were collected at each time point for further studies. Occurrence of cholecystolithiasis was annotated through macroscopic observation. All animal experiments were performed at Fundación Ciencia & Vida (Santiago, Chile), following the approved guidelines as dictated by the Institutional Animal Care and Use Committee from the same institution.

#### 2.1.2 Pharmacological interventions

In order to study the effects of aspirin and ezetimibe on the development of preneoplastic lesions and chronic inflammation, we designed a new animal cohort in which both lithogenic diet groups were treated with either aspirin or ezetimibe, starting at day 0. Low dose of aspirin (Aspirina®; Bayer Chile, Santiago, Chile) was supplemented to the drinking water at 30 mg·L^−1^dose every 2 weeks, as described previously [16]. The estimated dose of aspirin was 4 mg·kg^−1^considering an average daily water intake of 4 mL for every 30 g mouse, which corresponds to a human equivalent dose of 0.33 mg·kg^−1^and is three times the clinical human dose of 0.17 mg·kg^−1^. On the other hand, ezetimibe (Ezetrol®; Merck/MSD Chile, Santiago, Chile) was ground up and supplemented to the diet of both lithogenic groups at a dose of 50 mg·kg^−1^of diet, as previously described [14]. The estimated average dose of ezetimibe was 5–6 mg·kg^−1^·day^−1^per mouse, which corresponds to a human equivalent dose of 0.5 mg·kg^−1^. Hence, treated mice were grouped as follows: (a) lithogenic low‐cholesterol + aspirin (*n* = 16); (b) lithogenic low‐cholesterol + ezetimibe (*n* = 20); (c) lithogenic high‐cholesterol + aspirin (*n* = 20); and (d) lithogenic high‐cholesterol + ezetimibe (*n* = 22). Four to 12 mice from each group were euthanized at months 3 and 9 (Fig. 4A). Gallbladder, liver, spleen, and plasma samples were collected at both time points for further studies. Alike the previous mouse protocol, all of these animal experiments were performed at Fundación Ciencia & Vida, following the approved guideline as dictated by the Institutional Animal Care and Use Committee from the same institution.

### 2.2 Histopathological analysis

Liver samples were fixed in formalin and paraffin‐embedded. Gallbladder samples were first fixed in absolute EtOH for 24 h and next transferred to formalin fixation solution. Formalin‐fixed paraffin‐embedded (FFPE) tissues were stained with hematoxylin and eosin (H&E) solution. Two independent pathologists (N.G. and J.C.R.) examined the H&E cases using a blinded scoring system. Epithelial gallbladder alterations were tabulated after histopathological mapping of the entire gallbladder. The following parameters were analyzed: (a) thickness of the tunica muscularis; (b) epithelial hyperplasia of the mucosa; (c) fibrosis of the muscular layer; (d) metaplasia; (e) dysplasia; (f) presence and extension of hyaline bodies; (g) nuclear disorder; (h) atypia; and (i) anisokaryosis (variation in nuclei size). We also assessed inflammatory cell infiltrates, lymphocytes, and polymorphonuclear (PMN) cells, within the lesions. Inflammatory infiltrates were scored by two blinded pathologists (N.G. and J.C.R.), as follows: 0 = absent; 1 = low; 2 = moderate; and 3 = high.

Additionally, FFPE gallbladder sections were stained with pH 2.5 Alcian blue/periodic acid–Schiff (AB/PAS) mixture. Briefly, FFPE sections were stained with Alcian blue for 15 min and then washed in running tap water for 2 min. Subsequently, they were treated with periodic acid for 5 min, and washed and stained with Schiff's reagent for 10 min. Finally, the sections were washed and stained with hematoxylin solution.

Images were taken using Aperio Digital Pathology Slide Scanner AT2 (Leica Biosystems, Buffalo Grove, IL, USA).

### 2.3 Immunohistochemistry (IHC)

Formalin‐fixed paraffin‐embedded gallbladder sections (2 µm) were deparaffinized, rehydrated, and antigen‐retrieved by using pH 9.0 Tris/EDTA buffer, following antibody recommendations. Endogenous peroxidase activity was blocked for 15 min using 3% H_2_O_2_, and sections were incubated with indicated primary antibodies for 45 min at room temperature. Sections were then incubated with anti‐rabbit IgG secondary antibody for 30 min at room temperature (ImmPRESS™ Reagent; Vector Laboratories Inc., Burlingame, CA, USA). Next, sections were incubated with 33‐diaminobenzidine tetrachloride (DAB) and counterstained with hematoxylin. The following primary antibodies were used: F4/80 (ab111101, 1 : 100), CD3 (ab16669, 1 : 200), CD4 (ab183685, 1 : 1500), and CD8 (ab203035, 1 : 800), purchased from Abcam (Cambridge, UK). To determine the IHC score, a combined semiquantitative score was used for the assessment of immune cell infiltrates in FFPE sections, described elsewhere [17]. Two independent pathologists (X.G. and J.C.R) evaluated the following parameters: (a) abundance of inflammatory cells (0: absent; 1: low; 2: moderated; and 3: high) and (b) the intensity of the stain (0: absent; 1: weak; 2: moderated; and 3: strong). IHC score (from 0 to 9 values) was calculated by multiplication of these two parameters.

### 2.4 Transmission electron microscopy

Formalin‐fixed paraffin‐embedded gallbladder sections (0.5 µm) were deparaffinized by 10‐min immersion in xylol and hydrated through a progressive alcohol gradient comprising 10 min of 100% EtOH, 10 min of 70% EtOH, 10 min of 50% EtOH, and 10 min of water. Next, sections were fixed with 1% osmium tetroxide for 2 h at 4 °C. Samples were dehydrated with ascendant acetone gradient and embedded in Epon blocks. Sections were observed with a Philips Tecnai 12 (BioTWIN transmission electron microscope, Eindhoven, Netherlands).

### 2.5 Plasma collection and biochemical measurements

Blood samples were collected into EDTA‐coated Eppendorf tubes, and mixed and centrifuged for 15 min at 1500 ***g***at 4 °C. Next, plasma (supernatant) was transferred to a new tube and stored at −80 °C for further analysis. Triglycerols and total cholesterol were quantified by enzymatic‐colorimetric methods using an automated clinical analyzer (Metrolab 2300). Normal and pathological commercial serums were used for quality control assessment.

### 2.6 Flow cytometric analysis

Spleens were mechanically disrupted and filtered using a 70‐µmcell strainer. Cells were recovered by centrifugation and incubated with ACK lysis buffer for 5 min at room temperature. Then, cells were washed twice with PBS and resuspended in PBS supplemented with 2% of FBS [Life Technologies (Burlington, ONT, Canada) A31604]. Then, cells were counted in Neubauer chamber and 1 × 10^6^cells were stained with the antibodies listed in Table S1. All antibodies were used according to the manufacturer's guidelines. Myeloid and lymphoid immune gating strategies are shown in Fig. S1.

For intracellular staining, 1 × 10^6^cells were cultured for 12 h in Cell Stimulation Cocktail with protein transport inhibitors [eBioscience (ThermoFisher Scientific, Waltham, MA, USA) 00‐4975‐93]. Next, cells were stained with antibodies listed in Table S1. Cells were fixed and permeabilized with BD Cytofix/Cytoperm [BD Biosciences (San Jose, CA, USA) 554714] followed by the addition of intracellular antibodies. Each sample was autocontrolled by fluorescence minus one strategy. All samples were processed in BD LSRFortessa X‐20 cytometer (BD Biosciences), and data were analyzed in flowjoVX0.7 (BD Biosciences).

### 2.7 Statistical analysis

Statistical power analysis was calculated for the evaluation of the proportion of animals that presented metaplasia and dysplasia in each diet group in Fisher's exact test analysis. A good statistical power (> 80%) was obtained for metaplasia comparisons, and these results can be considered as conclusive. For dysplasia analysis, a weak statistical power (~ 20%) was obtained and these results are exploratory. Therefore, additional studies are warranted. Percentage of preneoplastic lesions in mice from the diet‐induced gallbladder preneoplasia model and drug intervention cohorts (aspirin and ezetimibe) were compared by Fisher's exact test. Animal and liver weights and liver/animal weight ratio were compared in the previously mentioned mouse cohorts by Kruskal–Wallis test analysis followed by Dunn's *post hoc*test. Total plasma cholesterol and triglycerol levels were also compared between these groups by the same approach.

Chronic inflammation was also evaluated in the diet‐induced gallbladder preneoplasia model and drug intervention cohorts (aspirin and ezetimibe) by H&E and IHC staining.

Hematoxylin and eosin scores of PMN cells and lymphocytes were compared between mice from different groups of both cohorts by Kruskal–Wallis test followed by Dunn's *post hoc*test. We also compared H&E scores of PMN cells and lymphocytes grouped by control (normal) or different epithelial alterations (hyperplasia, fibrosis, metaplasia, and dysplasia) by the same approach. Finally, we performed Fisher's exact test to determine whether chronic inflammation (staged by PMN cell and lymphocyte H&E scores) was associated with the development of metaplasia and dysplasia. The same analyses were performed for IHC scores of immune cell infiltrates (macrophages, and CD4+ and CD8 T+ cells), only in the diet‐induced gallbladder preneoplasia model.

All statistical analyses were performed using rprogramming environment in RStudio© version 1.0 (RStudio, Inc., Boston, MA, USA). Statistical significance (*P‐*value) was set at < 0.05.

## 3 Results

### 3.1 Lithogenic diets promote the development of gallbladder stones (cholecystolithiasis) and fatty liver disease in susceptible C57BL/6 mice

Previous studies have shown that lithogenic high‐cholesterol diets induce cholecystolithiasis in susceptible mice [8, 14, 15]. Consistent with these reports, our results showed that mice fed with lithogenic high‐cholesterol diet had higher plasmatic cholesterol levels than those fed with chow diet. Additionally, we observed higher plasmatic cholesterol levels in the lithogenic low‐cholesterol group than in chow diet mice, although this increase was less than the lithogenic high‐cholesterol group (Fig. S2A). This trend was not observed for triglycerol levels (Fig. S2A). All lithogenic mice (low‐ and high‐cholesterol) developed gallbladder stones from 1 to 9 months. Macroscopically at 1 month, we observed an increment in gallbladder size in both lithogenic diet groups compared to chow diet (Fig. 1B). All mice fed with either of the lithogenic diets presented various amounts of gallbladder stones of different sizes (Fig. 1C). Taken together, these results show that both lithogenic diets are sufficient to induce a significant increase in plasmatic cholesterol levels and to promote the onset of cholecystolithiasis. Interestingly, biliary crystals from the lithogenic diet groups differed in shape. While lithogenic high‐cholesterol mice developed the typical rectangular‐shaped cholesterol crystals (similar to humans), biliary crystals from lithogenic low‐cholesterol mice had amorphous shapes (Fig. 1D). In addition, liver weights and liver/body weight ratios were higher in lithogenic high‐cholesterol mice than in lithogenic low‐cholesterol and chow mice (Fig. S2B). Moreover, both lithogenic groups developed fatty liver disease (characterized by ballooning of hepatocytes and presence of lipid droplets) observed in H&E‐stained tissues (Fig. S2C).

### 3.2 Lithogenic diet induces the development of metaplasia and dysplasia in cholecystolithiasic mice

To study the relationship between cholecystolithiasis and development of gallbladder preneoplastic lesions, animals were exposed to lithogenic diet for 9 months. Although previous reports have studied the development of cholecystolithiasis under lithogenic diet [14, 15], none have studied the effect of cholecystolithiasis on the development of gallbladder preneoplastic lesions for extended periods of time. We further evaluated gallbladder epithelial lesions at months 1, 2, 3, and 9 in the three diet groups. Thickening of the tunica muscularis layer was observed in both lithogenic diets at all time points. Strikingly, we observed the presence of ramified glands (indicative of pseudopyloric metaplasia) in lithogenic high‐cholesterol and lithogenic low‐cholesterol mice at months 1 and 2, respectively (Fig. 1E). These structural changes were absent in the chow group. At month 3, 25% (1/4) of mice under lithogenic low‐cholesterol diet and over 50% (7/12) of mice under lithogenic high‐cholesterol diet developed metaplasia, which reached 100% of mice from both lithogenic groups at month 9 (Fig. 1F). Moreover, both lithogenic diets were significantly associated with the development of metaplasia (Table 1), indicating that lithogenic diet is a risk factor for gallbladder metaplasia development. Concurrent glandular hyperplasia and fibrosis of the muscular layer were observed in metaplastic gallbladders from both lithogenic groups (Fig. S3). At 9 months, presence of dysplasia was observed only in lithogenic high‐cholesterol group (Fig. 1E). Dysplastic lesions were characterized by nuclear atypia, anisokaryosis, loss of nuclear polarity, and atypical mitosis (Fig. S4). Five out of eight (62.5%) lithogenic high‐cholesterol mice developed this preneoplastic lesion, which was not found in chow and lithogenic low‐cholesterol group (Fig. 1F). Moreover, lithogenic high‐cholesterol diet was associated with the development of dysplasia (Table 1; *P* = 0.081 vs chow diet and *P* = 0.031 vs lithogenic low‐cholesterol diet). Despite the high percentage of dysplastic lesions, we did not observe development of invasive carcinoma at any time point.

**Table 1:** Association between diet and development of metaplasia and dysplasia at 9 months. Fisher's exact test analysis.

Diet	Metaplasia	No metaplasia	*P‐*value
Chow diet (*n* = 4)	0	4	
Litho‐low‐cholesterol diet (*n* = 6)	6	0	0.005*
Litho‐high‐cholesterol diet (*n* = 8)	8	0	0.002*

Diet	Dysplasia	No dysplasia	*P‐*value
Chow diet (*n* = 4)	0	4	
Litho‐low‐cholesterol diet (*n* = 6)	0	6	1.000
Litho‐high‐cholesterol diet (*n* = 8)	5	3	0.081

* Statistically significant association between each diet type and metaplasia/dysplasia development.

### 3.3 Presence of hyaline bodies in mice fed with lithogenic diets

We observed intracytoplasmic deposits in altered epithelial cells of both lithogenic diets, which were negative for AB/PAS staining, henceforth referred to as hyaline bodies (Fig. S5A). Unlike hyaline bodies, metaplastic cells were positive for AB/PAS staining, indicating the presence of goblet‐like cells expressing acid and neutral mucins (Fig. S5A). To study the composition of these negative AB/PAS deposits, we performed transmission electron microscopy on FFPE slides enriched for hyaline bodies. These cytoplasmic inclusions were osmiophilic and dark, indicating high affinity to fat (Fig. S5B). Therefore, we suggest that these hyaline bodies may correspond to accumulated lipids. We generated a score based on the extension of hyaline bodies within the tissue sections: 0 = absent; 1 = lower than 25% of cells; 2 = between 25% and 50% of cells; and 3 = higher than 50% of cells. We compared hyaline bodies' scores between histopathological lesions and normal tissues by Kruskal–Wallis/Dunn's *post hoc*test. Scores were significantly higher in hyperplasia, fibrosis, and metaplasia than in normal epithelium (Fig. S5C). Next, we determined the statistical relationship between epithelial lesions and presence of hyaline bodies in > 25% of cells (score > 1) by Fisher's exact test, which showed consistent results (Table S2). To the best of our knowledge, these hyaline bodies are not found in human gallbladder epithelial cells in the context of cholecystolithiasis.

### 3.4 Chronic inflammation is strongly associated with the development of gallbladder preneoplastic lesions

**Fig. 2:**
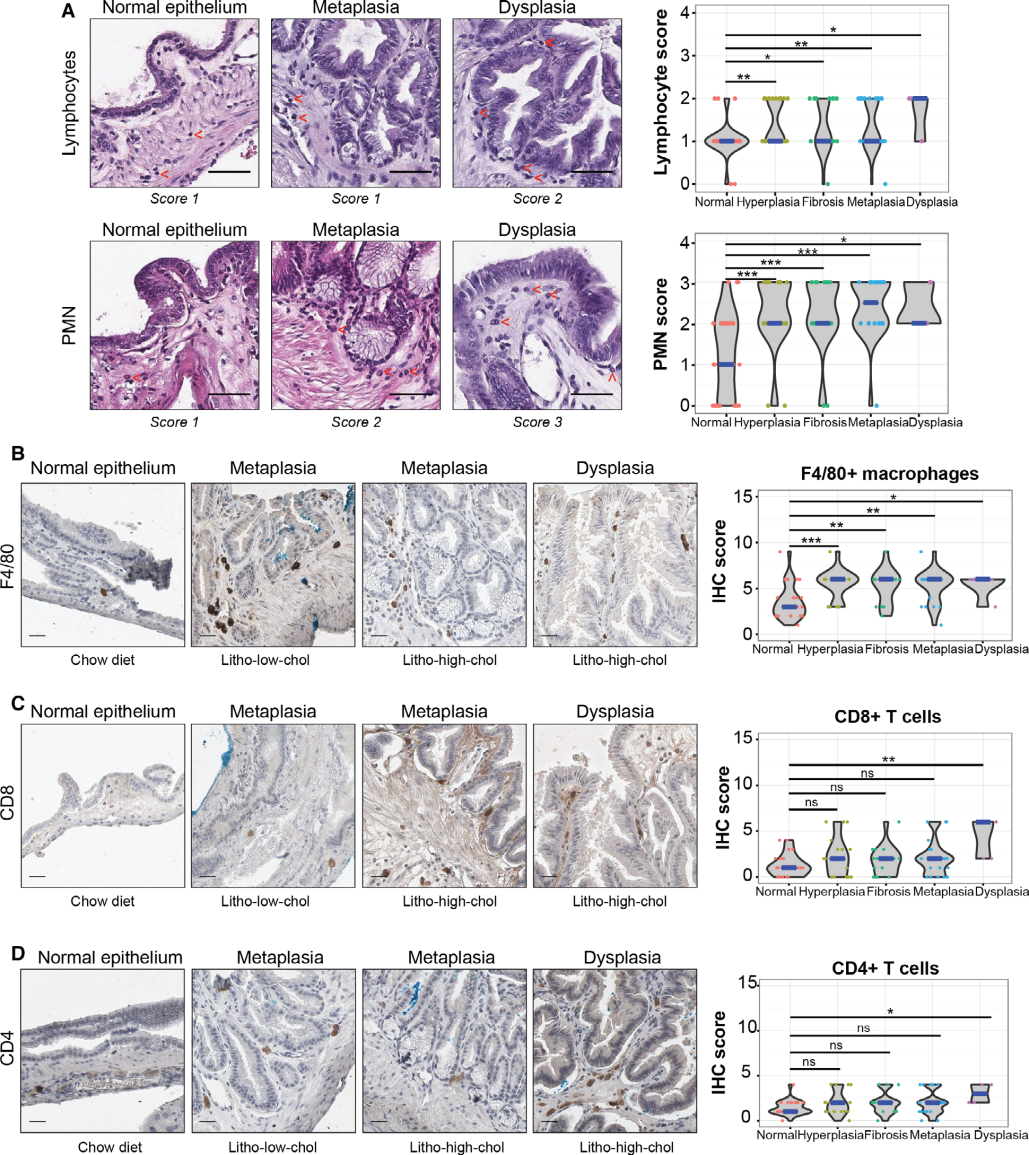
Leukocyte infiltration is higher in epithelial alterations than normal gallbladder epithelial samples. (A) Left: H&E‐stained gallbladder sections. Red arrows indicate lymphocytes and PMN cells (top and bottom panels, respectively) (scale bar, 50 µm). Right: Violin plots showing H&E scores for both cell types grouped by epithelial alterations (*n*sample = 34, 22, 20, 26, and 5 for normal epithelium, hyperplasia, fibrosis, metaplasia, and dysplasia; respectively). Lymphocytes and PMN infiltration are higher among altered epithelial samples than normal gallbladder epithelial samples. (B–D) Immunostaining (left) and IHC score (right) analysis of F4/80+ macrophages, CD8+ T cells, and CD4+ T cells (scale bar, 100 µm). In violin plots, the median is indicated in blue. Sample numbers for normal epithelium, hyperplasia, fibrosis, metaplasia, and dysplasia in each condition are as follows: 26, 17, 13, 20, and 5 for F4/80 + IHC score; 26, 20, 15, 23, and 5 for CD8+ IHC score; and 25, 19, 14, 22, and 4 for CD4+ IHC score, respectively. Multiple comparison analyses were performed with Kruskal–Wallis and Dunn's *post hoc*test. *P < 0.05, **P < 0.01, ***P < 0.001 and ns: not significant.

Metaplasia nearly always arises in tissues exposed to chronic injury or infections, hence undergoing continuous regeneration [18]. A common finding in human gallbladders with cholesterol stones is the presence of metaplasia and dysplasia in a context of chronic inflammation; characterized by fibrosis, wall thickening, and leukocyte infiltration [7]. To evaluate whether a similar inflammatory profile is present in this novel mouse model, we assessed the types of inflammatory cells in H&E‐stained gallbladder sections. We observed significantly higher H&E scores for lymphocytes and PMN cells in hyperplastic, fibrotic, metaplastic, and dysplastic areas compared to normal epithelia (Fig. 2A). We further evaluated leukocyte subpopulations by IHC. Significantly higher IHC scores for F4/80+ macrophages were found in hyperplasia, fibrosis, metaplasia, and dysplasia compared with normal epithelium (Fig. 2B). Consistent with observations in human samples, we also observed higher macrophage IHC scores in metaplasia [7]. Interestingly, we found a significant increment in IHC scores for CD8+ and CD4+ T cells in dysplasia compared to normal gallbladder (Fig. 2C,D). However, no differences were observed for CD3+ T cells (Fig. S6), suggesting a shift in the proportions of T‐cell subpopulations. Further, increment of PMN H&E scores and F4/80+ macrophage IHC scores was associated with the development of metaplasia, while increase of CD8+ T‐cell IHC scores was associated with dysplasia development (Tables S3‐S4). Here, we found that high IHC scores for innate immune cells (PMN and F4/80+ macrophages) were present at early stages (hyperplasia and metaplasia) and were sustained for 9 months, while adaptive immune cell scores (CD4+ and CD8+ T cells) increased at later stages (dysplasia). The prolonged presence of these two types of immune responses showed that chronic inflammation is concurrent with the development of epithelial lesions in mice with cholecystolithiasis.

### 3.5 Cholecystolithiasic mice display changes in splenic immune cell levels

Since the spleen is an important reservoir of macrophages and T cells in inflammatory immune responses, we evaluated changes in splenic immune cells in this animal model at months 3 and 9 by flow cytometry. We observed a global decrease of splenic CD4+ and activated CD8+ CD25+ T cells in mice with gallbladder epithelial lesions compared to normal mice. Interestingly, we found increased percentages of splenic regulatory T cells (CD4+ FoxP3+ IL‐10+) in mice with hyperplastic, fibrotic, metaplastic, and dysplastic gallbladders compared to normal gallbladder (Fig. S7A).

### 3.6 Lithogenic high‐cholesterol diet promotes local inflammation of the gallbladder as well as systemic inflammation

**Fig. 3:**
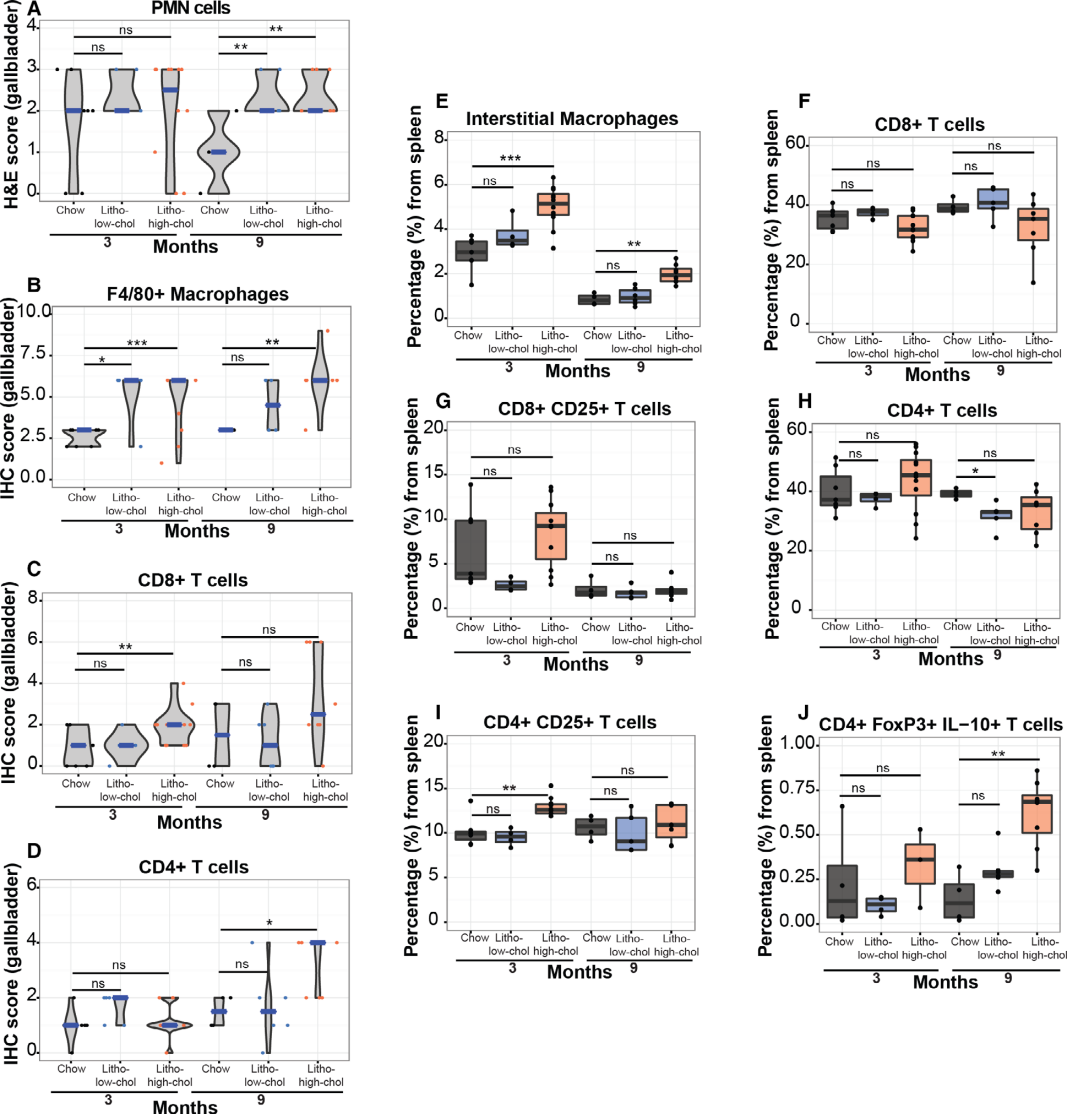
Local and systemic inflammation are triggered in both lithogenic diets. (A–D) Analysis of H&E score of PMN cells and IHC score of macrophages, CD8+ T cells, and CD4+ T cells in gallbladder tissues grouped by diet, at months 3 and 9. In violin plots, the median is indicated in blue. (E–J) Splenic levels (by flow cytometry) of macrophages, total CD8+ T cells, activated CD8+ T cells, CD4+ T cells, activated CD4+ T cells, and regulatory T cells grouped by diet, at months 3 and 9. *N*samples for myeloid cell population in the following groups at 3 and 9 months, respectively, are as follows: 7 and 4 for chow, 4 and 6 for litho‐low‐chol, and 12 and 8 for litho‐high‐chol. *N*samples for lymphoid cell population in the following groups at 3 and 9 months, respectively, are as follows: 7 and 4 for chow, 4 and 5 for litho‐low‐chol, and 12 and 7 for litho‐high‐chol. Regarding CD4+ FoxP3+ IL‐10+ cell population, *n*samples at 3 and 9 months, respectively, are as follows: 4 and 4 for chow group, 4 and 6 for litho‐low‐chol, and 3 and 8 for litho‐high‐chol. Multiple comparison analyses were performed with Kruskal–Wallis and Dunn's *post hoc*test. *P < 0.05, **P < 0.01, ***P < 0.001 and ns: not significant.

We evaluated the impact of each diet on immune cell H&E and IHC scores from the gallbladder at months 3 and 9. Significantly elevated PMN H&E scores were found in both lithogenic groups compared to chow diet at month 9 (Fig. 3A). We observed significant high IHC scores of F4/80 + macrophages, CD8 + T cells, and CD4+ T cells for months 3–9, 3, and 9, respectively, only in lithogenic high‐cholesterol compared to chow mice (Fig. 3B–D). Next, we sought to evaluate the impact of each diet on systemic inflammation by performing flow cytometric analysis of the spleens of mice from each group. We observed a significant high percentage of macrophages at months 3 and 9 and activated CD4+ CD25+ T cells at 3 months in lithogenic high‐cholesterol mice compared to the chow group (Fig. 3E,I). No differences were found in CD8+ T cells (Fig. 3F,G). Interestingly, splenic regulatory T cells were significantly increased in lithogenic high‐cholesterol mice compared to chow mice at 9 months (Fig. 3J), which may be an adaptive response to counteract an exacerbated inflammatory state. Additionally, we observed significantly higher levels of antigen‐presenting CD11b+ CD103+ dendritic cells at months 3 and 9 in lithogenic high‐cholesterol mice compared to chow mice (Fig. S7B). We did not observe significant differences for the other studied immune cell subpopulations (Fig. S7B). Taken together, these results suggest that a lithogenic diet with high cholesterol induces local (gallbladder) and systemic (spleen) immune response in this mouse model.

### 3.7 Effects of ezetimibe and aspirin on formation of gallbladder stones and cholesterol levels

**Fig. 4:**
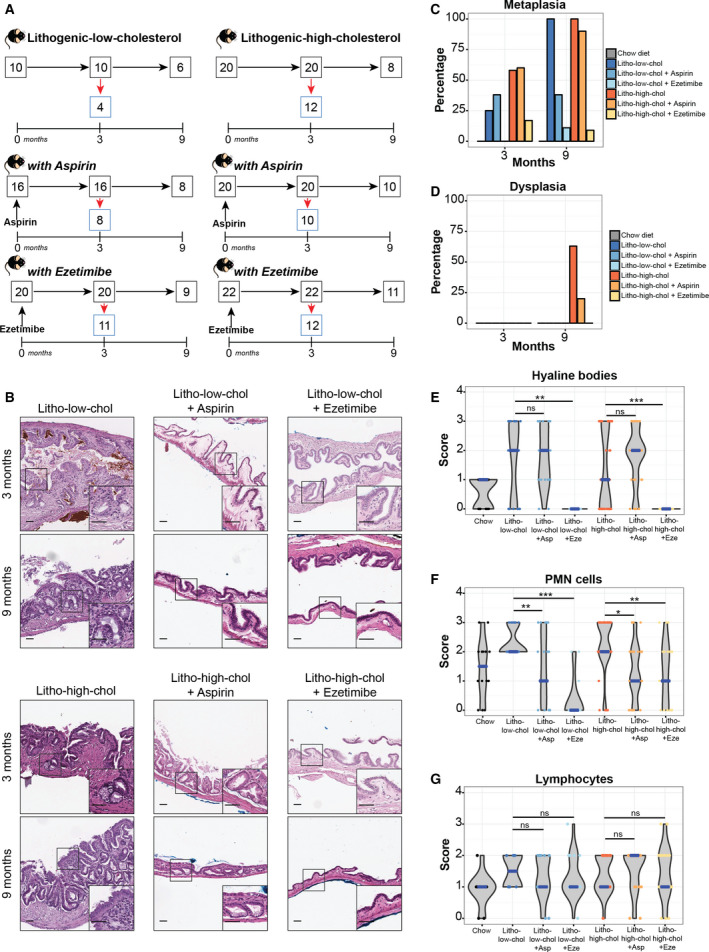
Effect of ezetimibe and aspirin on gallbladder preneoplasia and local inflammation. (A) Experimental design for the drug intervention mouse cohorts. Lithogenic low‐cholesterol (left) and lithogenic high‐cholesterol (right) either remained untreated (top panels) or treated with aspirin or ezetimibe (middle and bottom panels, respectively). Red arrows indicate number of mice that were euthanized at each time point, and black arrows indicate mice that remained for the following time point. (B) Histological changes observed after drug treatment. Both aspirin and ezetimibe reduced development of metaplasia on each group (onset magnifications showing normal‐like epithelium). Scale bar, 200 µm, and magnification scale bar, 50 µm. (C) Percentage of metaplasia induced by each diet and treatment. Number of animals with metaplasia of the total number in each group at 3 and 9 months, respectively: 1/4 and 6/6 for litho‐low‐chol, 3/8 and 3/8 for litho‐low‐chol + Asp, 0/11 and 1/9 for litho‐low‐chol + Eze, 7/12 and 8/8 for litho‐high‐col, 6/10 and 9/10 for litho‐high‐chol + Asp, and 2/12 and 1/11 for litho‐high‐chol + Eze. (D) Percentage of dysplasia induced by each diet and treatment. Dysplasia was only found in two groups: litho‐high‐chol (5/8) and litho‐high‐chol + Asp (2/10) at 9 months. Sample size for each group at each month was the same as indicated for metaplasia lesion. (E–G) Analysis H&E scores for hyaline bodies, PMN cells, and lymphocytes grouped by diet + treatment: chow, lithogenic low‐cholesterol (litho‐low‐chol), litho‐low‐chol + aspirin (litho‐low‐chol + Asp), litho‐low‐chol + ezetimibe (litho‐low‐chol + Eze), lithogenic high‐cholesterol (litho‐high‐chol), litho‐high‐chol + aspirin (litho‐high‐chol + Asp), and litho‐high‐chol + ezetimibe (litho‐high‐chol + Eze). Medians are indicated in blue. Sample numbers for each group at 3 and 9 months, respectively, are as follows: 7 and 9 for chow, 4 and 6 for litho‐low‐chol, 8 and 8 for litho‐low‐chol + Asp, 11 and 9 for litho‐low‐chol + Eze, 12 and 8 for litho‐high‐chol, 10 and 10 for litho‐high‐chol + Asp, and 12 and 11 for litho‐high‐chol + Eze. Multiple comparison analyses were performed with Kruskal–Wallis and Dunn's *post hoc*test. *P < 0.05, **P < 0.01, ***P < 0.001 and ns: not significant.

Given that gallbladder stones and chronic inflammation are two key factors that drive preneoplastic lesions, we tested the chemopreventive effect of two FDA‐approved drugs: (a) cholesterol absorption inhibitor ezetimibe and (b) anti‐inflammatory aspirin. We addressed the long‐term impact of ezetimibe and aspirin, separately, on the development of gallbladder preneoplastic lesions in a new cohort of mice fed with lithogenic low‐cholesterol or lithogenic high‐cholesterol diets. Experimental design is shown in Fig. 4A. Treatment with ezetimibe efficiently inhibited gallbladder stone formation in 100% of both lithogenic low‐cholesterol (20/20) and lithogenic high‐cholesterol mice (22/22), consistent with previous reports [14, 15]. Aspirin had no effects on the formation of stones: 87.5% (14/16) of lithogenic low‐cholesterol mice and 100% (20/20) of lithogenic high‐cholesterol mice treated with aspirin presented cholecystolithiasis. Ezetimibe significantly reduced total plasma cholesterol in lithogenic low‐cholesterol and lithogenic high‐cholesterol mice compared with nontreated groups, while aspirin treatment reduced plasma cholesterol only in the lithogenic high‐cholesterol group (Fig. S8A). Moreover, ezetimibe‐treated mice did not develop steatotic liver and showed decreased liver weight and liver/body weight ratio (Fig. S8B,C).

### 3.8 Ezetimibe prevents the onset of gallbladder preneoplastic lesions more effectively than aspirin

Treatment with aspirin or ezetimibe reduced the thickness of the gallbladder muscular layer in lithogenic low‐ and lithogenic high‐cholesterol mice compared to untreated mice (Fig. 4B). Moreover, aspirin reduced the development of metaplasia from 100% to 38% in lithogenic low‐cholesterol mice at 9 months (Fig. 4C; Table 2). On the other hand, ezetimibe greatly reduced metaplasia development in both lithogenic diets from 100% of animals to around 10% (Fig. 4C; Table 2). For dysplasia, while aspirin induced a nonsignificant reduction from 62.5% to ~ 20%, ezetimibe completely inhibited development of dysplasia at 9 months (Fig. 4D; Table 2). Moreover, the use of ezetimibe was significantly associated with the inhibition of metaplasia and dysplasia at 9 months (Table 2).

**Table 2:** Association between each treatment and the reduction of the development of metaplasia and dysplasia at 9 months. Fisher's exact test analysis.

Diet	Metaplasia	No metaplasia	*P‐*value
Litho‐low‐cholesterol diet (*n* = 6)	6	0	
Litho‐low‐cholesterol + aspirin diet (*n* = 8)	3	5	0.031*
Litho‐low‐cholesterol + ezetimibe diet (*n* = 9)	1	8	0.001*
Litho‐high‐cholesterol diet (*n* = 8)	8	0	
Litho‐high‐cholesterol + aspirin diet (*n* = 10)	9	1	1.000
Litho‐high‐cholesterol + ezetimibe diet (*n* = 11)	1	10	0.0001*

Diet	Dysplasia	No dysplasia	*P‐*value
Litho‐low‐cholesterol diet (*n* = 6)	0	6	
Litho‐low‐cholesterol + aspirin diet (*n* = 8)	0	8	1.000
Litho‐low‐cholesterol + ezetimibe diet (*n* = 9)	0	9	1.000
Litho‐high‐cholesterol diet (*n* = 8)	5	3	
Litho‐high‐cholesterol + aspirin diet (*n* = 10)	2	8	0.145
Litho‐high‐cholesterol + ezetimibe diet (*n* = 11)	0	11	0.005*

* Statistically significant association between each treatment and reduction of each preneoplastic lesion.

Furthermore, there was a significant decrease in hyaline bodies and PMN scores using ezetimibe (Fig. 4E,F), suggesting that the presence of hyaline bodies and PMN infiltration correlates with the development of preneoplastic lesions in this model. Aspirin reduced only PMN scores in both lithogenic diets (Fig. 4F). No differences in lymphocyte infiltration were observed between treatments (Fig. 4G).

### 3.9 Aspirin and ezetimibe generate changes at systemic inflammation

**Fig. 5:**
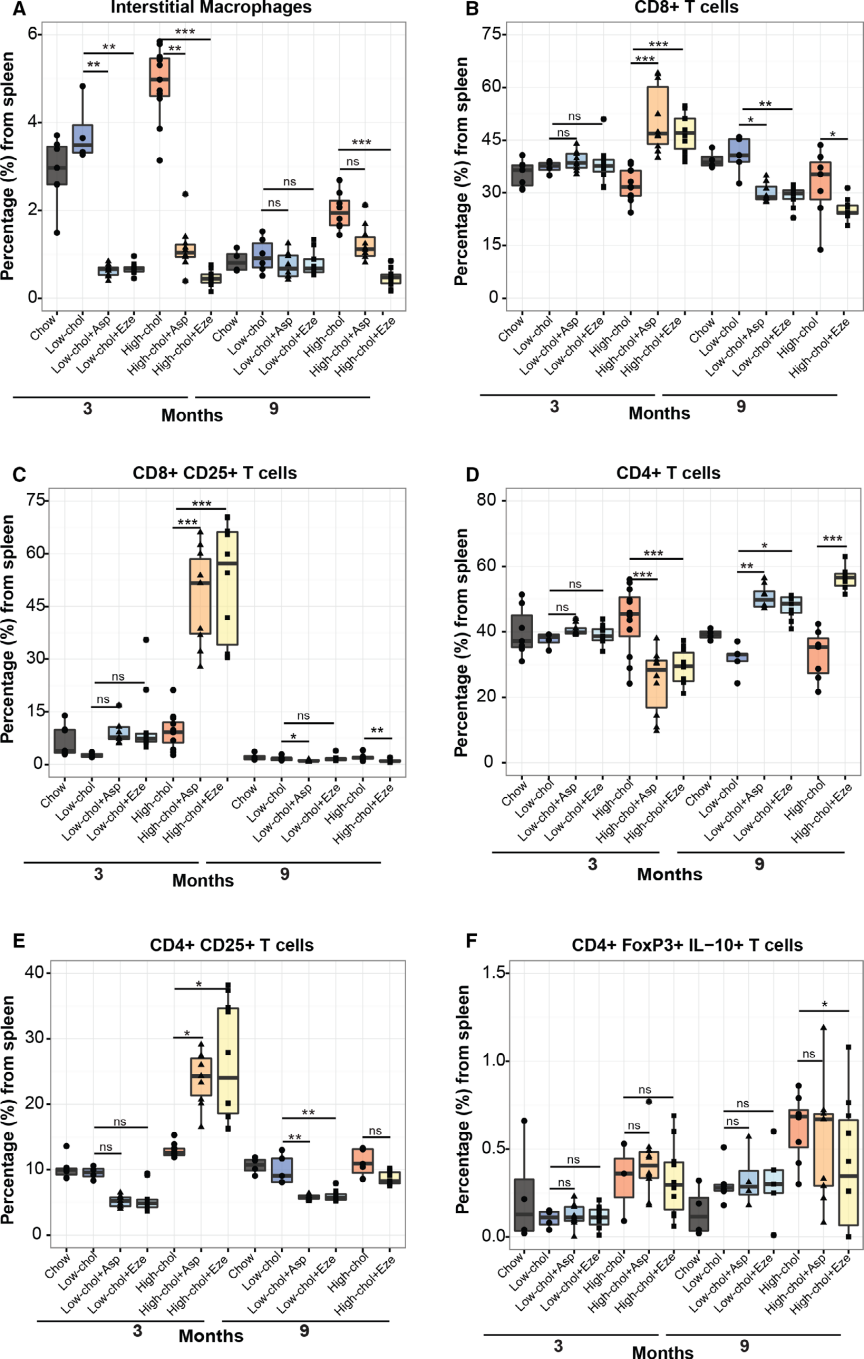
Effects of ezetimibe and aspirin on systemic inflammation. (A–F) Flow cytometric analysis of splenic populations of macrophages, total CD8+ T cells, activated CD8+ T cells, CD4+ T cells, activated CD4+ T cells, and regulatory T cells grouped by diet and treatment, at months 3 and 9. *N*samples for myeloid cell population in the following groups at 3 and 9 months, respectively, are as follows: 7 and 4 for chow, 4 and 6 for litho‐low‐chol, 9 and 8 for litho‐low‐chol + Asp, 11 and 9 for litho‐low‐chol + Eze, 12 and 8 for litho‐high‐chol, 10 and 10 for litho‐high‐chol + Asp, and 10 and 10 for litho‐high‐chol + Eze. *N*samples for lymphoid cell population in the following groups at 3 and 9 months, respectively, are as follows: 7 and 4 for chow, 4 and 5 for litho‐low‐chol, 9 and 8 for litho‐low‐chol + Asp, 11 and 9 for litho‐low‐chol + Eze, 12 and 7 for litho‐high‐chol, and 10 and 10 for litho‐high‐chol + Eze. Regarding CD4 + FoxP3+ IL‐10 + cell population, *n*samples at 3 and 9 months, respectively, are as follows: 4 and 4 for chow group, 4 and 6 for litho‐low‐chol, 3, 9, and 4 for litho‐low‐chol + Asp, 11 and 5 for litho‐low‐chol + Eze, 3 and 8 for litho‐high‐chol, 10 and 10 for litho‐high‐chol + Asp, and 10 and 10 for litho‐high‐chol + Eze. Multiple comparison analyses were performed with Kruskal–Wallis and Dunn's *post hoc*test. Of notice, high‐chol + Asp (9 months) in B‐E is missing due to experiment failure (*n* = 10 for 3 months). *P < 0.05, **P < 0.01, ***P < 0.001 and ns: not significant.

To assess the impact of aspirin and ezetimibe on systemic inflammation, we evaluated changes in splenic levels of immune cell subpopulations in mice from each treatment group. At month 3, both aspirin and ezetimibe treatment significantly reduced interstitial macrophage levels. Interestingly, at month 9, only ezetimibe significantly reduced interstitial macrophage levels in lithogenic high‐cholesterol mice (Fig. 5A). No significant effects were observed in mice from the lithogenic low‐cholesterol group at month 9. On contrary, we observed a significant increment of CD8+ T cells and activated CD8+ CD25+ T cells at 3 months in lithogenic high‐cholesterol mice treated with aspirin or ezetimibe compared to untreated lithogenic high‐cholesterol mice (Fig. 5B,C). At month 3, we observed a significant reduction in total CD4+ T cells, while activated CD4+ CD25+ T cells increased in treated lithogenic high‐cholesterol mice compared with untreated lithogenic high‐cholesterol mice (Fig. 5D,E). Surprisingly, we observed an inversion of this trend at month 9, where total CD4+ T cells were significantly increased in both drug treatments compared to untreated mice from the lithogenic low‐cholesterol group, while activated CD4+ CD25+ T cells decreased in both treatments in the lithogenic low‐ and lithogenic high‐cholesterol diet groups (Fig. 5D,E). Regulatory CD4+ FoxP3+ IL‐10+ T cells decreased at month 9 in lithogenic high‐cholesterol mice treated with ezetimibe (Fig. 5F). Additional splenic immune cell subpopulations are shown in Fig. S9.

## 4 Discussion

We have described that diet‐induced gallbladder stones trigger epithelial and inflammatory changes in mouse gallbladder that resembles several of the pathological features observed in human cholecystolithiasic gallbladders. Mice under lithogenic high‐cholesterol diet developed metaplasia in all animals and dysplasia in 62.5% of mice at 9 months. Noticeably, invasive carcinoma was not observed at any stage. To date, three GBC *in vivo*models have been described, mainly using transgenic mice [19, 20, 21]. However, none of these models recapitulate the metaplasia–dysplasia cancer sequence observed in humans. Since cholecystolithiasis is the main risk factor for GBC in humans, our model is a particularly promising platform to study and characterize early events driving gallbladder carcinogenesis, including epithelial alterations and chronic inflammation.

A major feature of our model is the fast and high onset of metaplasia, which was observed as early as 1 month upon starting the experiments and increased to 100% of the cohort at 9 months. Metaplasia is commonly observed and regarded as a preneoplastic lesion in several epithelial cancers including esophagus, stomach, pancreas, and gallbladder [7, 10]. Although the exact mechanisms underlying the development of metaplasia are not fully understood, it is known that onset of this lesion is closely associated with prolonged exposure to tissue damage and inflammation. We found high levels of PMN cells and macrophages at early stages of the preneoplastic process, indicating a rapid activation of the innate immune response to tissue injury. Although in physiological conditions neutrophils and macrophages are essential for the elimination of necrotic cells and wound repair, excess of these cells favors tumor development through activation of the IL‐6/Stat3 signaling pathway, which promotes cellular proliferation [22]. In addition, an increase of these inflammatory cells has been reported in Barrett's‐like metaplasia of the esophagus and intestinal metaplasia of the stomach [23, 24]. Interestingly, we observed a strong association between high PMN cell and macrophage scores with the development of metaplasia. Strikingly, we found high infiltration of PMN cells during the development of the diet‐induced gallbladder preneoplasia mouse model, which are crucial for the aggregation of calcium and cholesterol crystals to produce gallbladder stones [25]. We also found elevated CD8+ T‐cell infiltration, which were associated with the development of dysplasia. Taken together, our data suggest that chronic inflammation progresses in parallel with the epithelial alterations observed early during gallbladder carcinogenesis.

Although GBC is a relatively rare malignancy worldwide, high prevalence is found mainly in South‐East Asia (Thailand and India) and South America (Chile and Bolivia). Chileans with admixed Native American ancestry have an elevated risk for developing gallbladder stones due to the presence of D19H variant in hepatic cholesterol transporter ABCG8. In consequence, admixed Chileans with this genetic variant are prone to have cholesterol‐saturated bile, a condition that favors development of cholecystolithiasis [26, 27]. In Chile, where GBC represents the third cause of cancer death in women, cholecystectomy is indicated as a preventive strategy to reduce GBC rates in all adults aged between 35 and 49 years with cholecystolithiasis [28]. Although preventive cholecystectomy has been readily implemented in Chile since 2006, there has been sustained decrease in GBC mortality since earlier in 2000 [29]. Therefore, it seems that reduction in GBC mortality may be due to additional factors aside from cholecystectomy. Moreover, several negative effects have been ascribed to preventive cholecystectomy in patients from different populations. These include higher prevalence of nonalcoholic fatty liver disease, weight gain/obesity, and metabolic syndrome [30]. Therefore, new strategies for secondary prevention are needed.

We proposed ezetimibe as a potential chemopreventive drug to preclude the development of GBC. It has been previously reported that the use of ezetimibe in cholecystolithiasic mice inhibits gallbladder stone formation [14, 15]. However, the long‐term impact of ezetimibe on the development of gallbladder preneoplastic lesions has not been described. In this study, we showed that treatment with ezetimibe dramatically reduced the rate of metaplasia and dysplasia. These results strongly support that inhibition of cholesterol absorption and subsequent global decrease of plasma cholesterol effectively reduce the impact of a lithogenic state on gallbladder stone formation and development of gallbladder preneoplastic lesions. Hence, it would be interesting to test ezetimibe as a chemopreventive strategy in high‐risk populations for GBC. Additionally, we tested the anti‐inflammatory effects of low‐dose aspirin on the development of gallbladder preneoplastic lesions. This drug has been previously reported to reduce overall cancer incidence and mortality [31, 32, 33]. In esophageal cancer, which follows a metaplasia–dysplasia cancer sequence similar to GBC, aspirin effectively reduces the risk of high‐grade dysplasia and esophageal adenocarcinoma among patients with Barrett´s metaplasia [34]. Its low cost and wide availability make aspirin an interesting candidate for chemoprevention in GBC. Aspirin use has been previously associated with decreased risk of cholangiocarcinoma in a case–control study [35], but the effects of this drug on the development of gallbladder preneoplastic lesions have not been characterized. In our study, aspirin treatment did not decrease gallbladder stone formation, consistent with what has been observed in humans [7]. However, during aspirin treatment, we observed a partial reduction in metaplasia at 9 months only in the lithogenic low‐cholesterol group. This result was accompanied by a significant reduction of infiltrative PMN cells in the gallbladder of both lithogenic diet mouse groups. These evidences suggest a link between the inflammatory axis targeted by aspirin and the development of metaplasia. Indeed, inflammatory signaling has been implicated in the generation of metaplasia in other organs. The cytokines RANTEs and TNF‐α can induce acinar‐to‐ductal metaplasia in pancreas through activation of NF‐κB [36]. In esophagus, overexpression of IL‐1β is sufficient to induce metaplasia and dysplasia in mice, thus showing that inflammatory signaling plays a relevant role in the induction of metaplasia [23]. Additional studies are warranted to describe which of these immune mechanisms are involved in the development of gallbladder preneoplastic lesions.

In summary, our results show that both lithogenic diets lead to high plasmatic cholesterol levels in wild‐type C57BL/6 mice. It has been reported that high plasmatic cholesterol levels correlate with increased cholesterol saturation of the bile, which favors the formation of cholesterol crystals and gallbladder stones [14]. The persistence of these stones promotes sustained epithelial damage, which is translated to epithelial alterations encompassing hyperplasia, fibrosis, metaplasia, and dysplasia. Metaplasia corresponds to the first preneoplastic lesion in this context of sustained injury. At this stage, we observed high infiltration of PMN cells and F4/80+ macrophages. Innate immune cells secrete pro‐inflammatory cytokines and chemokines to activate and recruit additional inflammatory cell populations [37]. Consistently, at later stages (dysplasia), we observed high infiltration of CD4+ and CD8+ T cells (adaptive immune response). During the development of preneoplastic lesions, we also observed high levels of interstitial macrophages in the spleen, indicating systemic inflammation in cholecystolithiasic mice. Thus, lithogenic diet likely triggers a systemic inflammatory state that warrants further exploration in the future.

Exacerbated inflammatory response and preneoplasia development can be reverted by the use of the FDA‐approved drug ezetimibe, which inhibits cholesterol absorption [14]. Ezetimibe causes a significant reduction of the plasmatic cholesterol levels, indicating lower cholesterol availability in lithogenic mice. Consequently, gallbladder stones are not generated. In the absence of gallbladder stones, the epithelial lining of the gallbladder is not subjected to sustained injury. Thus, no adaptive response is required and metaplasia does not develop. Substantiating these claims, we observed that neither local nor systemic inflammatory response cascades were triggered in lithogenic mice treated with ezetimibe, as illustrated by the reduction of PMN cell infiltration in the gallbladder and lower levels of interstitial macrophages in the spleen, respectively. On the other hand, low‐dose aspirin reduced infiltration of PMN cells in the gallbladder and splenic interstitial macrophages. However, this dose of aspirin only reduced the proportion of metaplasia in lithogenic low‐cholesterol mice. Low dose of aspirin was insufficient to revert the development of metaplasia and dysplasia in lithogenic high‐cholesterol mice. This low dose of aspirin was previously reported by Cyrus *et al*. [16]. In this study, the authors observed reduced infiltration of macrophages in aortic lesions in an atherosclerosis mouse model. Additionally, a slightly higher dose (1.5‐fold higher) effectively reduced hepatic Cox2 expression levels in C57BL/6 mice [38].

**Fig. 6:**
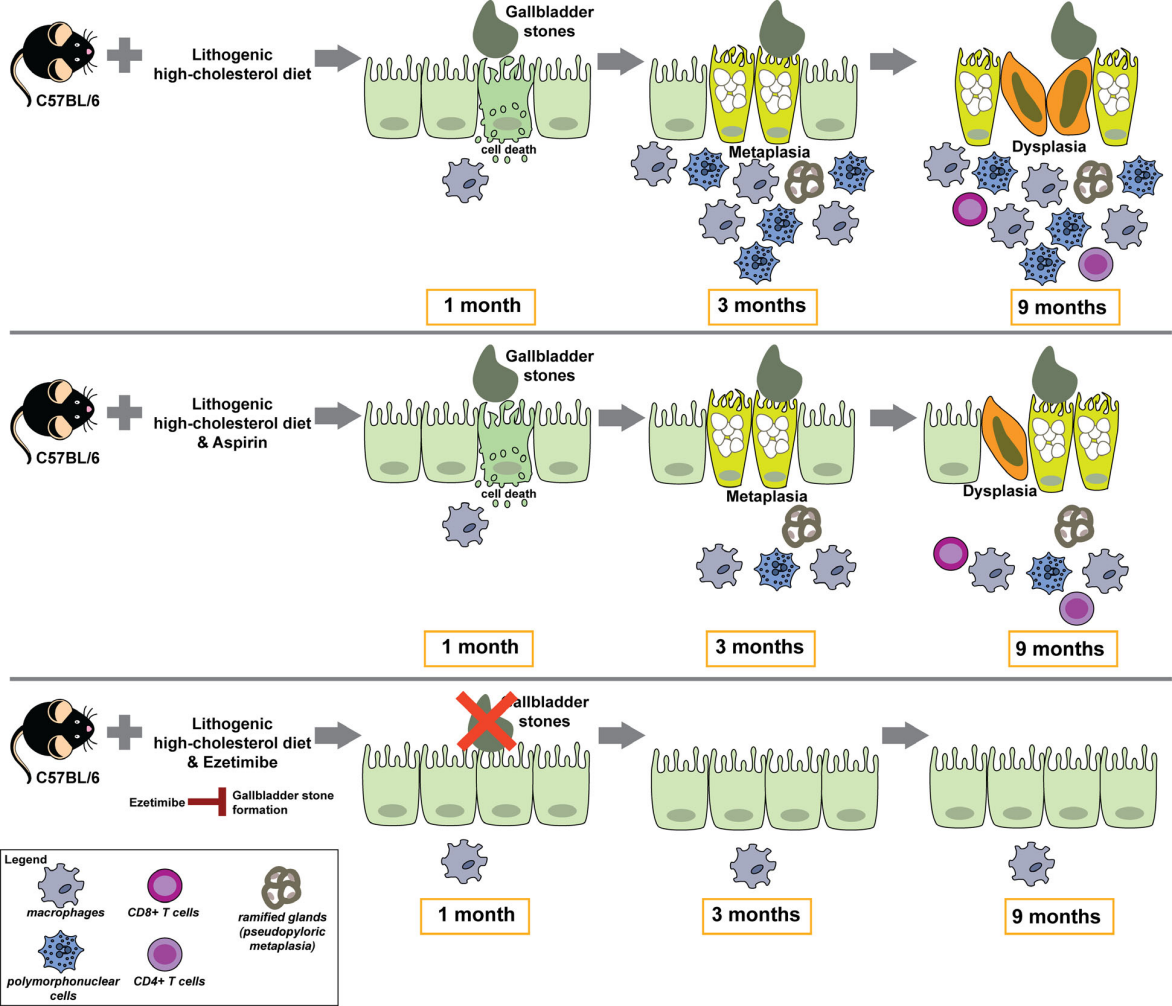
Gallbladder stones promote inflammation and progression of gallbladder preneoplasia associated in C57BL/6 mice fed with lithogenic high‐cholesterol diet. Mice fed with lithogenic high‐cholesterol diet (upper panel) develop gallbladder stones at 1 month. At 3 months, tissue injury is translated into the development of metaplasia accompanied with high infiltration of macrophages and PMN cells. At later stages, dysplasia is developed with emergence of CD8+ and CD4+ T‐cell infiltrates. Inhibition of inflammation by aspirin (middle panel) did not reduce the development of metaplasia or dysplasia in lithogenic high‐cholesterol mice. Conversely, inhibition of gallbladder stone formation by ezetimibe (lower panel) prevented the development of inflammation, metaplasia, and dysplasia.

Contrary to what we observed for ezetimibe treatment, low‐dose aspirin showed a weak effect in the prevention of gallbladder preneoplastic lesions. The inability of this drug to prevent the onset of metaplasia and dysplasia may be explained, at least in part, due to the low dosage of aspirin and the persistence of gallbladder stones in the experimental group, which perpetuate tissue injury and inflammation, and favor the development of gallbladder preneoplastic lesions. The effects of higher doses of aspirin should be explored in this novel gallbladder preneoplasia mouse model in future studies. In Fig. 6, we summarize the main histopathological changes and immune cells involved in gallbladder preneoplasia progression and the effects of aspirin and ezetimibe on the gallbladder epithelium.

## 5 Conclusions

In the present study, we have developed and characterized a novel mouse model of gallbladder preneoplasia. Our work demonstrated that lithogenic high‐cholesterol diet induces the development of cholecystolithiasis, chronic inflammation, and preneoplastic lesions in wild‐type C57BL/6 mice. Importantly, this murine model recapitulates the metaplasia–dysplasia sequence observed in humans and is a promising platform for preneoplasia research. Moreover, inhibition of cholecystolithiasis by ezetimibe completely prevented the inflammatory response and the onset of metaplasia and dysplasia, supporting that ezetimibe is an important candidate to be considered for chemoprevention testing in high‐risk GBC populations.

## Conflict of interest

The authors have no conflicts of interest to declare.

## Author contributions

LR, LL‐G, PG, CB, JFM, MA, JAE, and JCR conceived the study and designed the experiments. LR, LL‐G, FG, and JV performed *in vivo*experiments. LR performed tissue stains, IHC experiments, and tissue imaging. NM‐D performed flow cytometric experiments and designed gating strategy. NG and JCR performed histopathological analysis of complete mapped gallbladders. XG and JCR scored the immunostains. NS performed cholesterol and triglycerols quantification of plasma samples. LR performed data analysis, statistical analyses, and designed the figures. IAW performed statistical power analysis (calculation and interpretation), writing assistance, and language edition. The manuscript was written by LR and JAE and critically reviewed by PG, CB, IAW, CF, AMK, JFM, MA, and JCR. All authors read and approved the final manuscript.

## Supporting information


**Table S1**. List of antibodies used for Flow Cytometry.
**Table S2**. Association between epithelial lesion and hyaline bodies H&E scores.
**Table S3**. Association between H&E and IHC scores of inflammatory infiltrates with the development of metaplasia.
**Table S4**. Association between H&E and IHC scores of inflammatory infiltrates with the development of dysplasia.
**Fig. S1**. Flow cytometry workflow.
**Fig. S2**. Increment of plasmatic total cholesterol and development of steatotic liver in both lithogenic groups.
**Fig. S3**. Gallbladder hyperplasia and fibrosis in lithogenic mice.
**Fig. S4**. Lithogenic‐high‐cholesterol mice developed dysplasia at 9 months.
**Fig. S5**. Both lithogenic mice developed hyaline bodies.
**Fig. S6**. Levels of T cells do not change in epithelial gallbladder alterations.
**Fig. S7**. Systemic levels of splenic immune cells in gallbladder preneoplasia.
**Fig. S8**. Effect of ezetimibe and aspirin on plasmatic levels of cholesterol and triglycerides and liver histology.
**Fig. S9**. Systemic levels of splenic immune cells in mice treated with aspirin and ezetimibe.

## Data Availability

All data are available in the main manuscript and in the Supporting information.
