# Supplementary material for: Evaluation of the chemopreventive potentials of ezetimibe and aspirin in a novel mouse model of gallbladder preneoplasia
Source: Mol Oncol. 2020 Sep 17;14(11):2834–52. doi: 10.1002/1878-0261.12766 (PMC7607176; doi:10.1002/1878-0261.12766)
Supplement: Supplementary file 1 — Table S1. List of antibodies used for Flow Cytometry. Table S2. Association between epithelial lesion and hyaline bodies H&E scores. Table S3. Association between H&E and IHC scores of inflammatory infiltrates with the development of metaplasia. Table S4. Association between H&E and IHC scores of inflammatory infiltrates with the development of dysplasia. Fig. S1. Flow cytometry workflow. Fig. S2. Increment of plasmatic total cholesterol and development of steatotic liver in both lithogenic groups. Fig. S3. Gallbladder hyperplasia and fibrosis in lithogenic mice. Fig. S4. Lithogenic‐high‐cholesterol mice developed dysplasia at 9 months. Fig. S5. Both lithogenic mice developed hyaline bodies. Fig. S6. Levels of T cells do not change in epithelial gallbladder alterations. Fig. S7. Systemic levels of splenic immune cells in gallbladder preneoplasia. Fig. S8. Effect of ezetimibe and aspirin on plasmatic levels of cholesterol and triglycerides and liver histology. Fig. S9. Systemic levels of splenic immune cells in mice treated with aspirin and ezetimibe. [file MOL2-14-2834-s001.pdf]

Table S1. List of antibodies used for Flow Cytometry.

Antibody	Company	Catalogue No.	Conjugate	Staining type
CD11b	BD Biosciences	553311	PE: phycoerythrin	Myeloid
CD103	BD Biosciences	563637	PerCPCy5.5: peridinin chlorophyll protein cyanine 5.5	Myeloid
CD24	BD Biosciences	563450	BV 711: Brilliant Violet 711	Myeloid
Ly6G	BD Biosciences	551460	FITC: fluorescein isothiocyanate	Myeloid
IA/IE	BD Biosciences	743872	BV 605: Brilliant Violet 605	Myeloid
CD64	BD Biosciences	558539	Alexa Fluor® 647	Myeloid
CD11c	BD Biosciences	558079	PECy7: phycoerythrin cyanine 7	Myeloid
TCRβ	BD Biosciences	553172	PE: phycoerythrin	Lymphoid
CD4	BD Biosciences	553051	APC: allophycocyanin	Lymphoid
CD8	BD Biosciences	553030	FITC: fluorescein isothiocyanate	Lymphoid
CD25	Biolegend	102026	APCCy7: allophycocyanin cyanine 7	Lymphoid
TCRβ	BD Biosciences	553171	FITC: fluorescein isothiocyanate	Intracellular
IL-10	BD Biosciences	564083	BV 650: Brilliant Violet 650	Intracellular
FoxP3	BD Biosciences	560408	PE: phycoerythrin	Intracellular

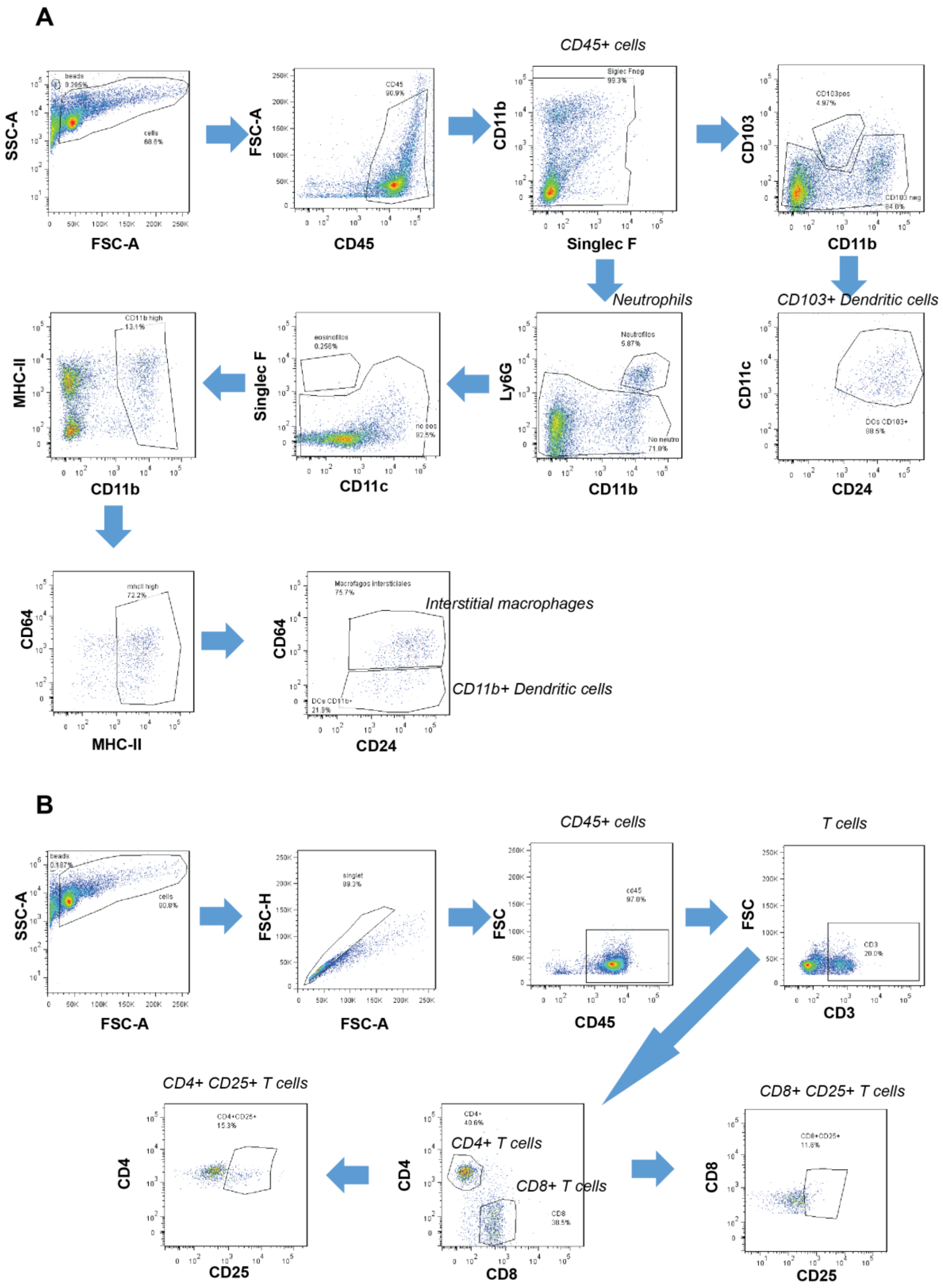

Figure S1. Flow cytometry workflow. (A) myeloid strategy and (B) lymphoid strategy.

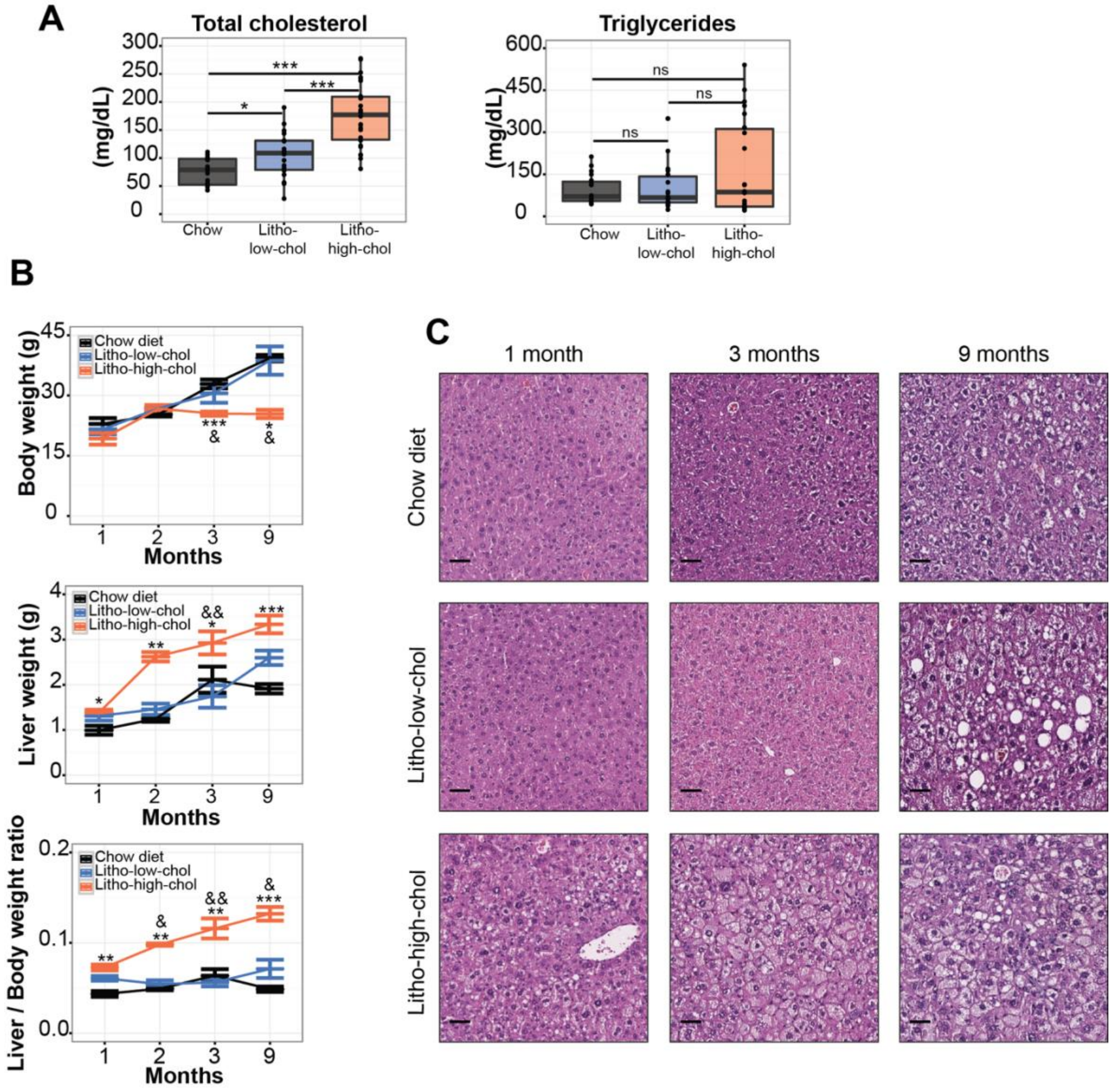

Figure S2. Increment of plasmatic total cholesterol and development of steatotic liver in both lithogenic groups. (A) Total plasma cholesterol and triglycerides levels (mg/dL) in each diet. Multiple comparison analyses were performed with Kruskal Wallis and Dunn's post hoc test. *P<0.05, **P<0.01, ***P<0.001 and ns: not significant. (B) Body and liver weights (g) and liver/body weight ratio in each diet. Multiple comparisons analyses were performed with Kruskal Wallis and Dunn's post hoc test: *P<.05, **P<.01, ***P<.001 in chow diet vs litho-high-cholesterol; &P<.05 and &&P<.01 for litho-low-cholesterol and litho-high-cholesterol comparisons. (C) Representative images showing the histological changes in the liver induced by each diet (scale bar, 100 µm).

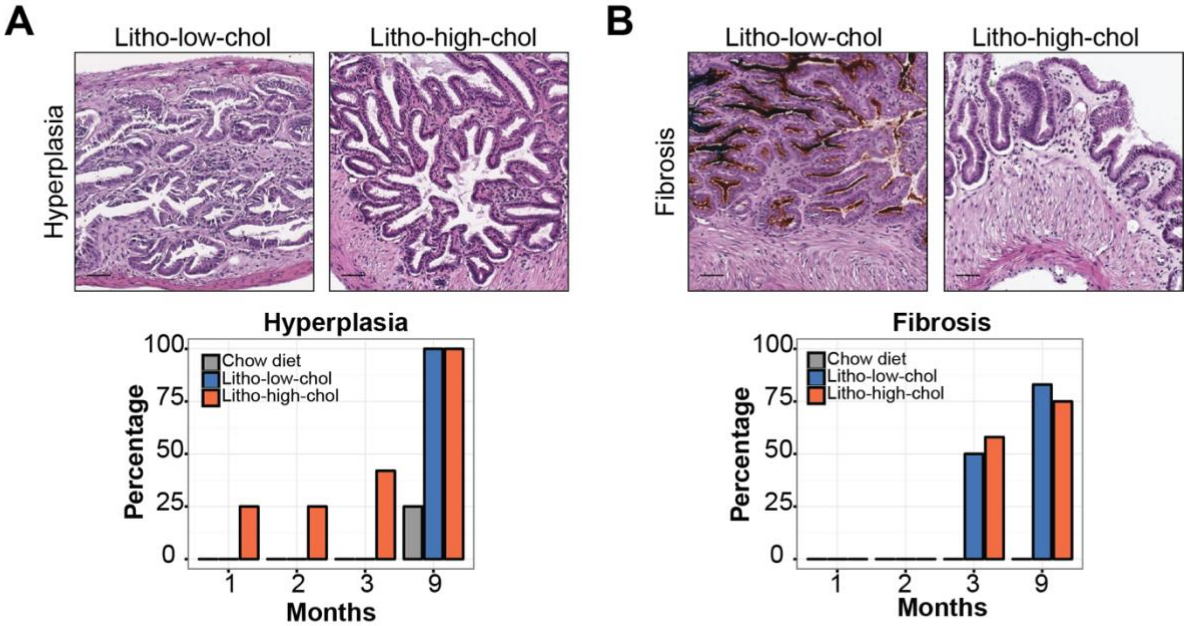

Figure S3. Gallbladder hyperplasia and fibrosis in lithogenic mice. (A-B) Representative images (bottom panel) of hyperplasia and fibrosis in litho-low-chol and litho-high-chol groups and percentage (low panel) of these alterations in each diet type and month (scale bar, 100 µm).

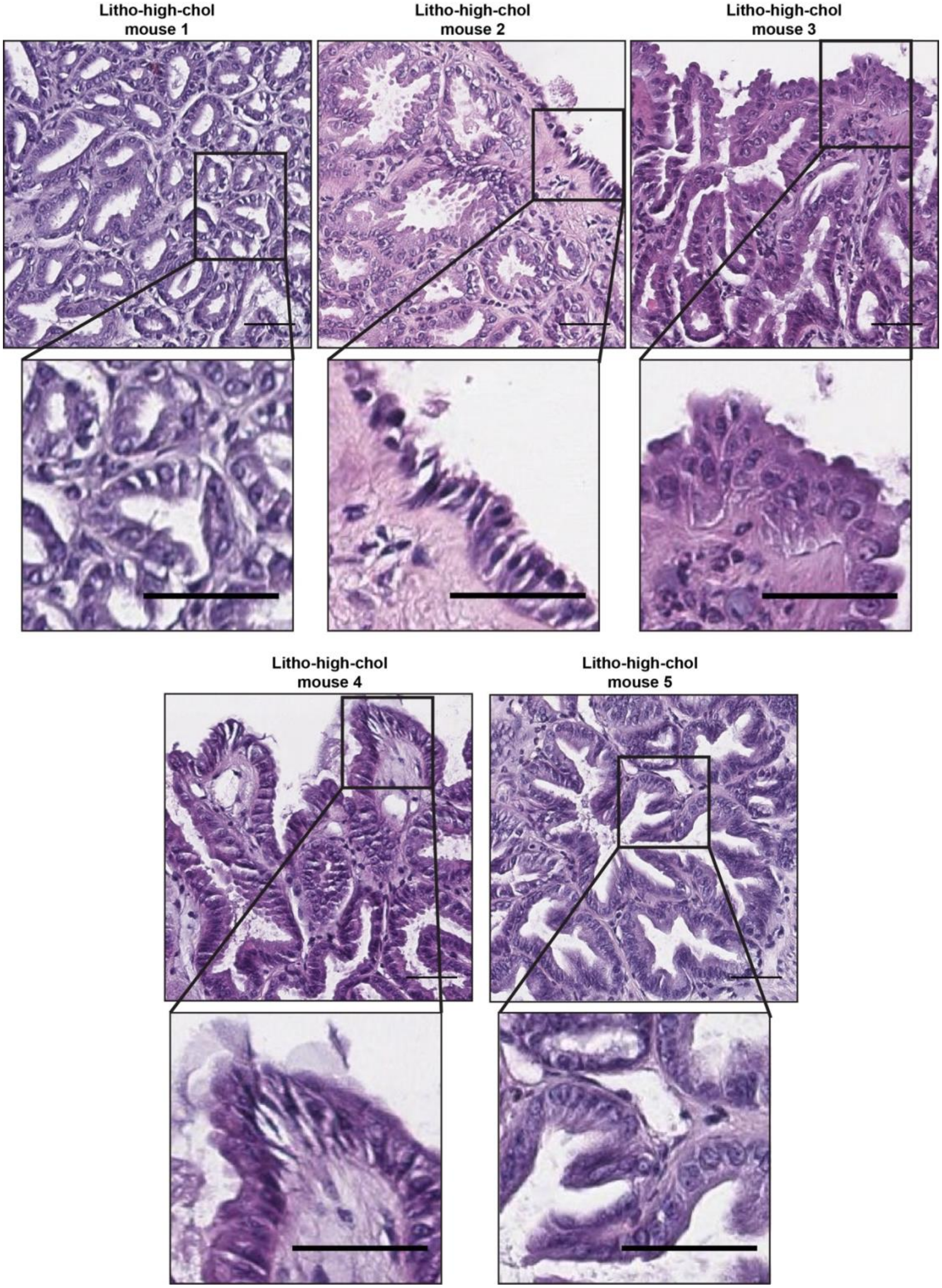

Figure S4. Lithogenic-high-cholesterol mice developed dysplasia at 9 months. Representative H&E images for dysplasia in lithogenic-high-cholesterol mice are shown (scale bar, 50 µm). Magnification onsets indicates nuclear alterations.

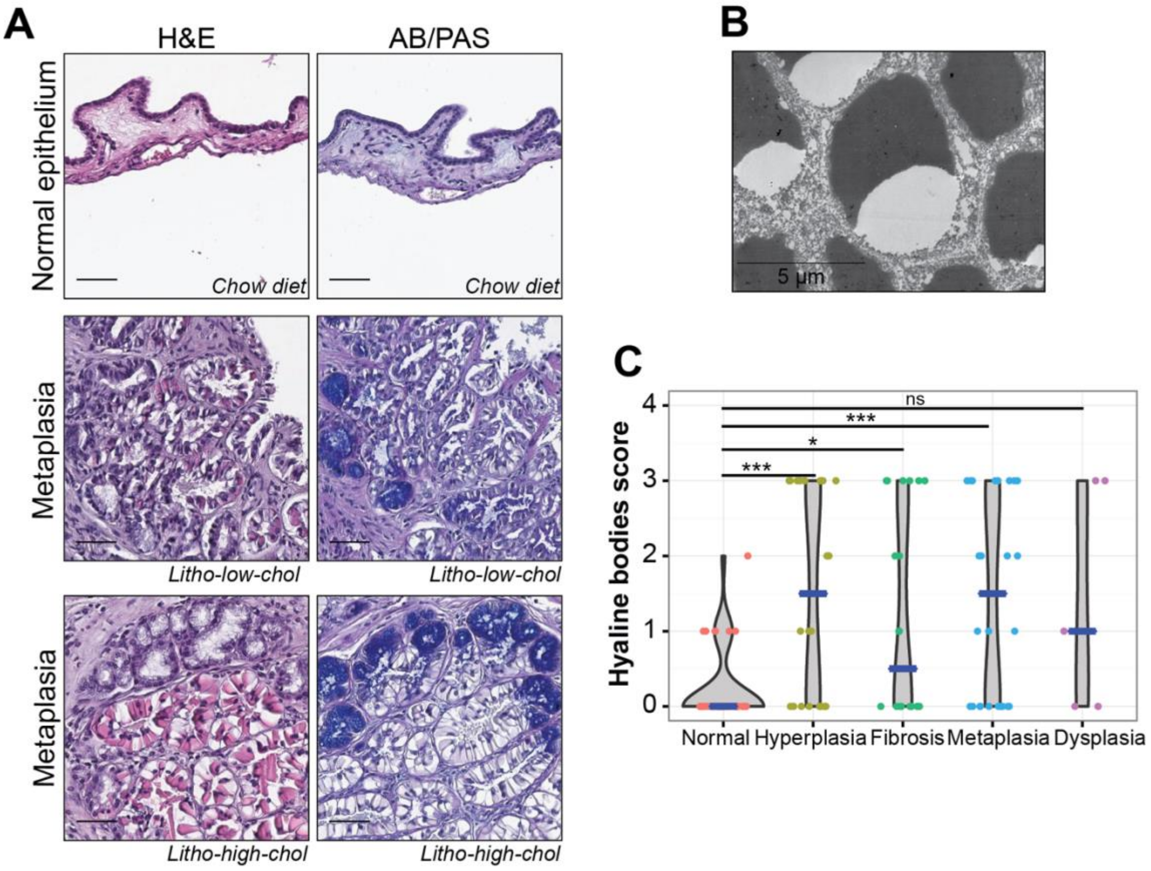

Figure S5. Both lithogenic mice developed hyaline bodies. (A) Alcian-blue/Periodic Acid Schiff (AB/PAS) staining showing acid mucins (stained blue) present in metaplastic lesions (scale bar, 100 µm). (B) Transmission electron microscopy image of hyaline bodies. (C) H&E scores of hyaline bodies in samples grouped by epithelial alterations and compared against normal gallbladder epithelium. The median is indicated in blue. Multiple comparisons analysis was performed with Kruskal Wallis and Dunn's post hoc test. **P*<0.05, ***P*<0.01, ****P*<0.001 and ns: not significant.

Table S2. Association between epithelial lesion and hyaline bodies H&E scores.

**Parameter**	**Epithelium type**	**H&E score**		***P-value***
		**Score ≤ 1**	**Score > 1**	
Hyaline bodies (n=108 cases)	Normal	33	1	
	Hyperplasia	11	11	0.0002*
	Fibrosis	12	8	0.0028*
	Metaplasia	13	13	0.0001*
	Dysplasia	3	2	0.0690

Fisher's exact test analysis. Asterisk indicates statistically significant association between each gallbladder epithelial alteration with hyaline bodies H&E score.

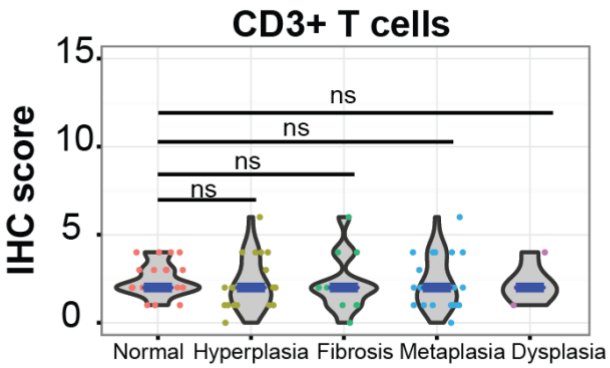

Figure S6. Levels of T cells do not change in epithelial gallbladder alterations. IHC score analysis of T cells. Median is indicated in blue. Multiple comparisons analysis was performed with Kruskal Wallis and Dunn's post hoc test. *P<0.05, **P<0.01, ***P<0.001 and ns: not significant.

Table S3. Association between H&E and IHC scores of inflammatory infiltrates with the development of metaplasia.

Inflammatory cell	H&E or IHC score	Metaplasia	No Metaplasia	*P-value*
Lymphocytes (H&E score) n=65	0	1	3	
	1	14	32	1.000
	2	11	4	0.117
	3	0	0	1.000
PMN (H&E score) n=65	0	1	12	
	1	0	9	1.000
	2	12	14	0.029*
	3	13	4	0.0002*
F4/80 (IHC score) n=48	0-2	1	9	
	3-4	3	15	1.000
	6 and 9	15	5	0.001*
CD3 (IHC score) n=50	0-2	15	19	
	3-4	5	10	0.542
	6 and 9	1	0	0.457
CD4 (IHC score) n=51	0-2	17	29	
	3-4	5	0	0.011*
	6 and 9	0	0	1.000
CD8 (IHC score) n=52	0-2	16	25	
	3-4	4	4	0.699
	6 and 9	3	0	0.073

Fisher's exact test analysis. Asterisk indicates statistically significant association between each inflammatory cell type and metaplasia development.

Table S4. Association between H&E and IHC scores of inflammatory infiltrates with the development of dysplasia.

Inflammatory cell	H&E or IHC score	Dysplasia	No Dysplasia	*P-value*
Lymphocytes (H&E score) n=65	0	0	4	
	1	0	46	1.000
	2	5	10	0.530
	3	0	0	1.000
PMN (H&E score) n=65	0	0	13	
	1	0	9	1.000
	2	3	23	0.538
	3	2	15	0.492
F4/80 (IHC score) n=48	0-2	0	10	
	3-4	1	17	1.000
	6 and 9	4	16	0.272
CD3 (IHC score) n=50	0-2	4	30	
	3-4	1	14	1.000
	6 and 9	0	1	1.000
CD4 (IHC score) n=51	0-2	2	44	
	3-4	2	3	0.043*
	6 and 9	0	0	1.000
CD8 (IHC score) n=52	0-2	2	39	
	3-4	0	8	1.000
	6 and 9	3	0	0.0008*

Fisher's exact test analysis. Asterisk indicates statistically significant association between each inflammatory cell type and dysplasia development.

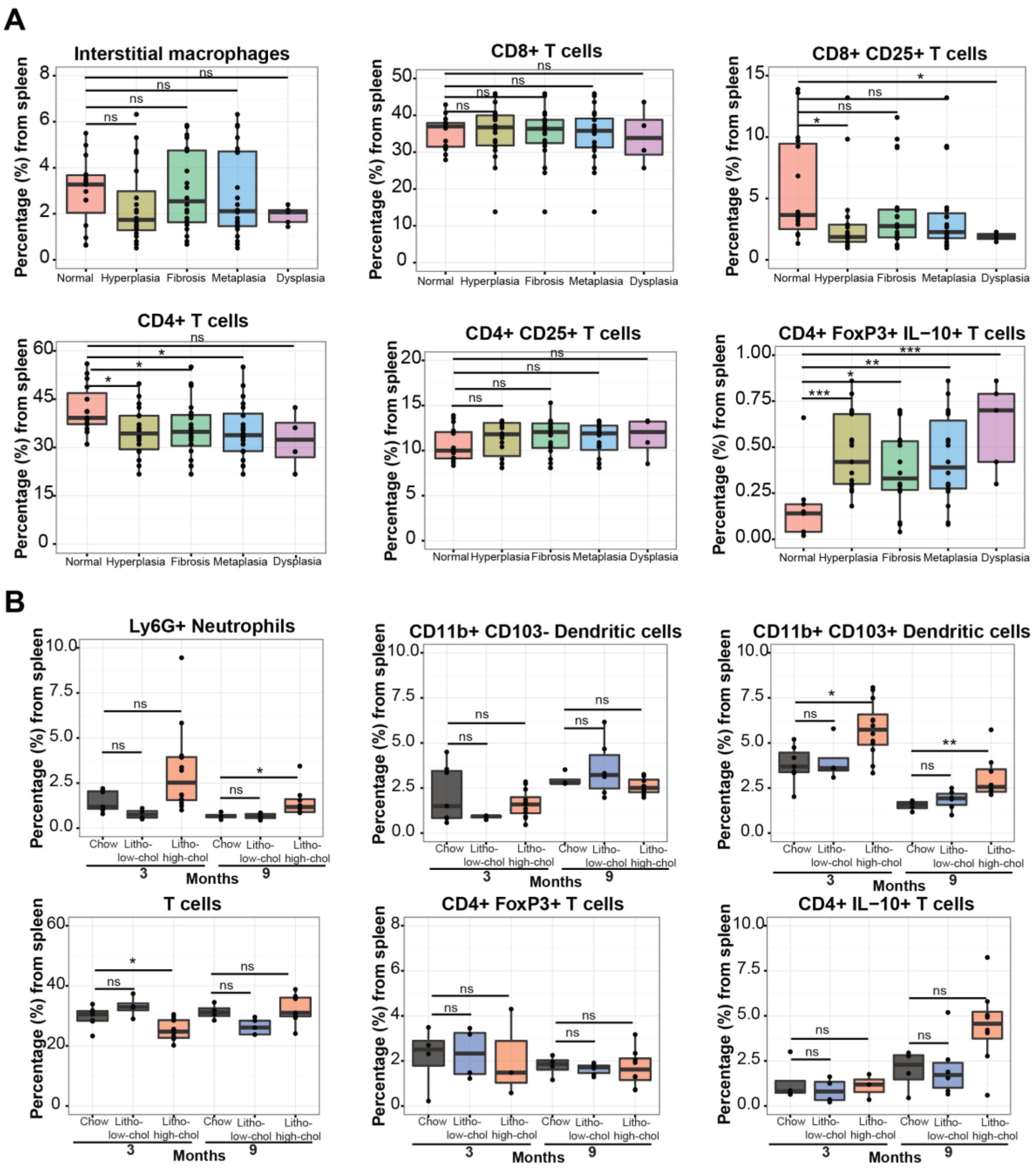

Figure S7. Systemic levels of splenic immune cells in gallbladder preneoplasia. (A) Splenic levels of macrophages, total CD8+, CD4+ T cells, activated CD8+ T and CD4+ cells and regulatory T cells in mice grouped by gallbladder epithelial alterations. (B) Percentage of splenic immune cells grouped by each diet. Multiple comparison analysis with Kruskal Wallis and Dunn's post hoc test. *P<0.05, **P<0.01, ***P<0.001 and ns: not significant.

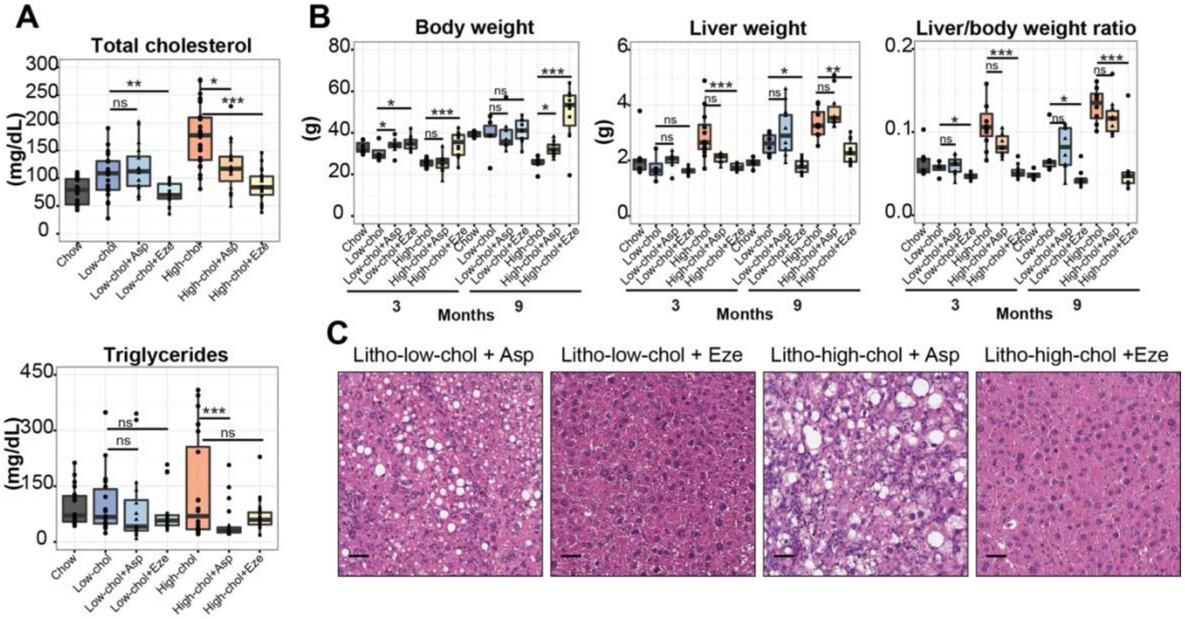

Figure S8. Effect of ezetimibe and aspirin on plasmatic levels of cholesterol and triglycerides and liver histology. (A) Total cholesterol and triglycerides levels (mg/dL) in each diet and treatment. (B) Body and liver weights (g) and liver/body weight ratio in each diet and treatment. Multiple comparisons analyses were performed with Kruskal Wallis and Dunn's post hoc test. *P<0.05, **P<0.01, ***P<0.001 and ns: not significant. (C) Representative images showing the histological changes in the liver grouped by each treatment (scale bar, 100 µm).

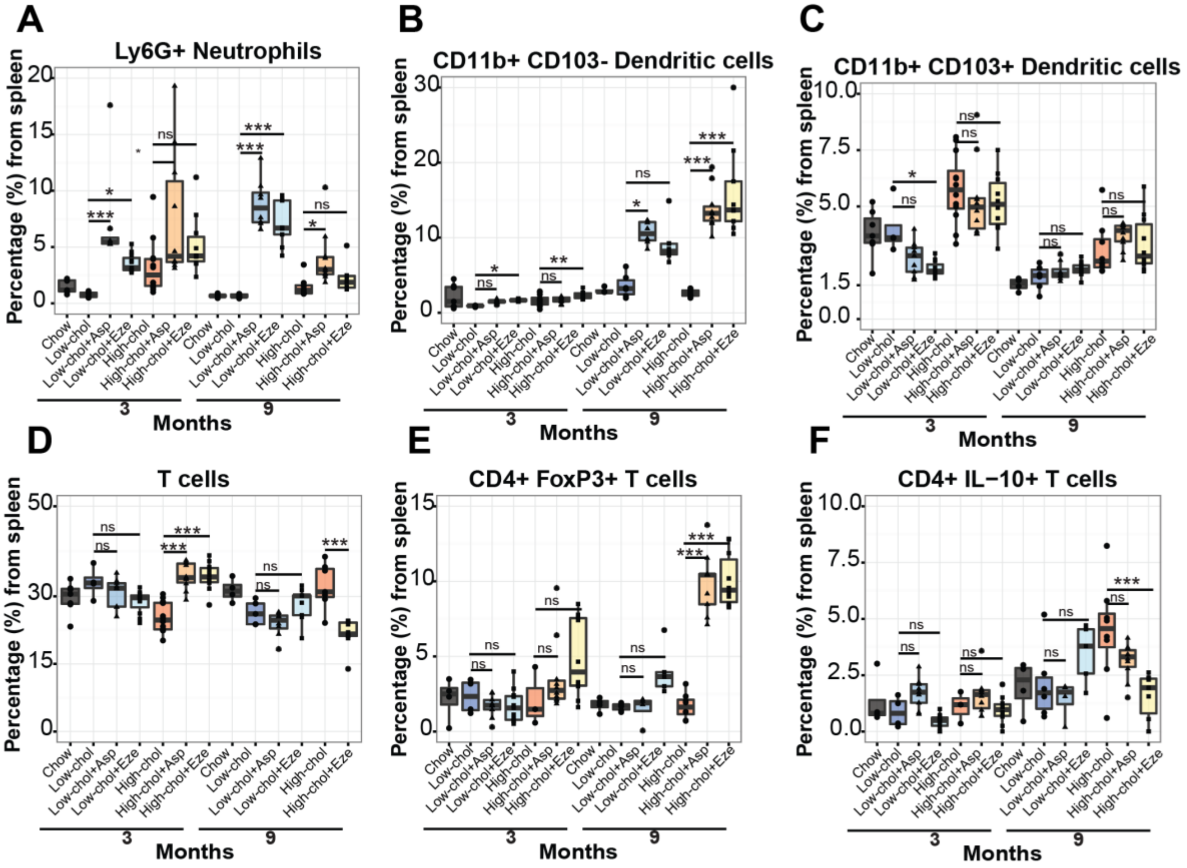

Figure S9. Systemic levels of splenic immune cells in mice treated with aspirin and ezetimibe. Percentage of splenic immune cells in each diet and treatment. Multiple comparison analyses were performed with Kruskal Wallis and Dunn's post hoc test. Of notice, High-chol + Asp in D is missing due to experiment failure. **P*<0.05, ***P*<0.01, ****P*<0.001 and ns: not significant.
